# Analysis of Autonomous Penetration Testing Through Reinforcement Learning and Recommender Systems

**DOI:** 10.3390/s25010211

**Published:** 2025-01-02

**Authors:** Ariadna Claudia Moreno, Aldo Hernandez-Suarez, Gabriel Sanchez-Perez, Linda Karina Toscano-Medina, Hector Perez-Meana, Jose Portillo-Portillo, Jesus Olivares-Mercado, Luis Javier García Villalba

**Affiliations:** 1 Instituto Politecnico Nacional, ESIME Culhuacan, Mexico City 04440, Mexico; amorenor2300@alumno.ipn.mx (A.C.M.); alhernandezsu@ipn.mx (A.H.-S.); gasanchezp@ipn.mx (G.S.-P.); ltoscano@ipn.mx (L.K.T.-M.); hmperezm@ipn.mx (H.P.-M.); jportillop@ipn.mx (J.P.-P.); jolivares@ipn.mx (J.O.-M.); 2 Group of Analysis, Security and Systems (GASS), Department of Software Engineering and Artificial Intelligence (DISIA), Faculty of Computer Science and Engineering, Office 431, Universidad Complutense de Madrid (UCM), Calle Profesor José García Santesmases, 9, Ciudad Universitaria, 28040 Madrid, Spain

**Keywords:** penetration testing, reinforcement learning, recommender systems

## Abstract

Conducting penetration testing (pentesting) in cybersecurity is a crucial turning point for identifying vulnerabilities within the framework of Information Technology (IT), where real malicious offensive behavior is simulated to identify potential weaknesses and strengthen preventive controls. Given the complexity of the tests, time constraints, and the specialized level of expertise required for pentesting, analysis and exploitation tools are commonly used. Although useful, these tools often introduce uncertainty in findings, resulting in high rates of false positives. To enhance the effectiveness of these tests, Machine Learning (ML) has been integrated, showing significant potential for identifying anomalies across various security areas through detailed detection of underlying malicious patterns. However, pentesting environments are unpredictable and intricate, requiring analysts to make extensive efforts to understand, explore, and exploit them. This study considers these challenges, proposing a recommendation system based on a context-rich, vocabulary-aware transformer capable of processing questions related to the target environment and offering responses based on necessary pentest batteries evaluated by a Reinforcement Learning (RL) estimator. This RL component assesses optimal attack strategies based on previously learned data and dynamically explores additional attack vectors. The system achieved an F1 score and an Exact Match rate over 97.0%, demonstrating its accuracy and effectiveness in selecting relevant pentesting strategies.

## 1. Introduction

With the continuous advancement of technology, cybersecurity has become crucial for protecting digital assets against threats, vulnerabilities, malicious artifacts, and other sophisticated cyber risks that impact their environment [1]. In this context, underpinned by the principles of Confidentiality, Integrity, and Availability (CIA), offensive cybersecurity is proposed as a range of dynamic techniques to robustly assess whether the applied controls and policies are effectively maintained within the targeted infrastructure [2].

In accordance with the National Institute of Standards and Technology (NIST) Special Publication (SP) 800-115, Technical Guide to Information Security Testing and Assessment [3], pentesting constitutes a fundamental component of offensive security. Through the execution of tests that simulate active attacks, mimicking the real behavior of a malicious actor, vulnerabilities in various information assets can be assessed, either manually or automatically. This process ultimately facilitates the identification of the most effective defense-response strategies.

**Figure 1:**
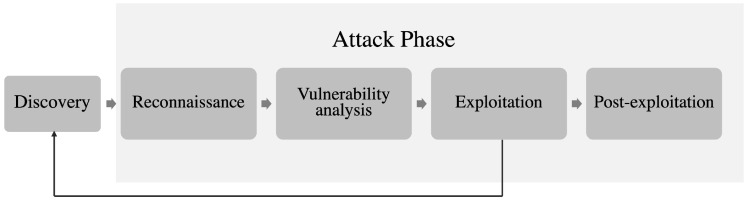
Phases of pentesting execution according to the best practices guide from SANS [4]. Note that the stages of reconnaissance, vulnerability analysis, exploitation, and post-exploitation are designated as attack phases, as these activities are actively engaged during the exercise. Additionally, the cycle may repeat if lateral movement opportunities arise during the exploitation stage.

While various methodologies and techniques for pentesting exist, the SysAdmin, Audit, Networking, and Security (SANS) Institute [4] has articulated a set of refined steps to conduct a comprehensive audit. This process begins with target identification, also referred to as the discovery phase, and proceeds through service reconnaissance, vulnerability analysis, exploitation, post-exploitation activities, and the generation of findings reports. Figure 1 illustrates a summary of these stages.

In this context, the backbone of the pentesting process lies in the careful design of tests, as the environments in which they are conducted often operate under a black-box premise. This means the analyst has only minimal knowledge of the environment and must dynamically uncover the relevant assets according to a structured reconnaissance and execution plan. This poses a constant challenge, as environments are typically dynamic and unpredictable, requiring a high level of expertise, finely calibrated tools, and substantial time windows, which frequently turns into a race between detection and mitigation due to the critical nature of the assets involved [5].

As argued in [6], while the expertise of the pentester and the adaptability of tools to agile methodologies enhance the design of test batteries based on key infrastructure components of the target environment, no approach can definitively anticipate the complexity or duration of the exercise. This unpredictability may, in turn, increase the likelihood of false positives stemming from unexplored gaps, unsuccessful proofs of concept, or imprecisions in testing.

Conversely, to address the previously mentioned challenges, there has been a growing interest in applying ML techniques within the pentesting design and execution process. This approach seeks a more automated, precise, and efficient perspective adaptable to various domains, specifically in the planning and attack phases of testing, thereby enabling experts to more comprehensively cover the targeted exploitation objectives [7].

Although various branches of ML have applied different algorithms to classify, cluster, or predict aspects of the pentesting process, Reinforcement Learning (RL) has distinguished itself by its ability to rapidly adapt to diverse reconnaissance surfaces and construct attack vectors in a more realistic manner. This adaptability allows RL to broaden its reach toward new horizons of exploration and exploitation in vulnerable scenarios, redefining pentesting practices [8].

The literature has thoroughly examined the benefits and challenges of using RL in cybersecurity, highlighting its ability to adapt to constantly evolving offensive and defensive technologies [9,10]. However, relying exclusively on an RL agent in pentesting is challenging due to the dynamic nature of environments and the exponential growth in tests required to optimize reconnaissance and attack phases. This challenge presents a prohibitive computational problem of order O(SAT), where *S* represents the target state space, *A* denotes the set of test actions, and *T* is the time horizon for executing potential attacks. The combination of these factors significantly increases the learning time and the trajectory required to find an optimal solution in vulnerable environments [8,11].

As indicated in [12], this situation has led pentesters, despite the wide availability of ML and RL tools, to continue relying on established databases such as the Common Vulnerability and Exposures (CVE) and the National Vulnerability Database (NVD). These resources allow them to identify vulnerable artifacts and focus on the proof-of-concept (PoC) tests needed to validate findings.

In this context, according to [13], the continuous pentesting process should rely on a balance between the estimation provided by the RL algorithm, the accuracy of intelligent testing, and expert validation, ensuring that results align with the environment requirements. Defining a general space for target attributes within the environment a finite domain for detecting vulnerabilities is essential. Calibrating the scope of the RL agent based on the probable number of tests is also crucial to prevent excessive generalization during the exploratory process. Additionally, consolidating results through pentester monitoring is fundamental to ensuring the quality of the procedure.

Within this framework, the present study addresses the limitations of current RL approaches in pentesting by proposing a resource-efficient architecture that maintains effectiveness while aligning closely with the skills of pentesters. The proposed model not only monitors all ecosystem components but also guides the analyst in precisely identifying the necessary tests and executing them optimally within a Question–Answer (QA) recommendation system (RS).

This approach leverages the synergy between RL and the text transformer BERT (Bidirectional Encoder Representations from Transformers) [14]. RL emulates the pentesting process within the desired domain and feeds a QA-based RS with tuples of question, context, and answer, enabling the pentester to consult and optimize the design, identification, and assessment of vulnerabilities. This integration allows the RL agent to learn from the evaluation environment and generate contextually relevant, actionable recommendations designed to guide the effective implementation of offensive tests, thereby avoiding system overload.

The structure of this manuscript is organized as follows: Section 2 examines related works that have explored traditional RL approaches, Quality Learning (QL), Deep Learning (DL) combined with RL (DRL), and hybrid models that manage one or more estimators or algorithms depending on the type of environment and the intended scope of exploitation for pentesting activities. Section 3 describes the methods and materials employed in developing the proposed RL system for QA-based recommendations within the pentesting process, hereafter referred to as BERT QA RL + RS, which is an acronym for Reinforcement Learning plus BERT plus Recommender System. Subsequently, Section 4 evaluates and discusses the results of RL+BERT+RS and compares them with other state-of-the-art studies. Finally, Section 6 presents the conclusions and suggests possible directions for future research.

## 2. Related Work

In [15], one of the first works to employ a traditional RL approach, the Intelligent Automated Penetration Testing (IAPT) approach is described. This approach utilizes a solution model based on the Partially Observable Markov Decision Process (POMDP), where an agent seeks the most optimal path to exploit vulnerabilities and earn rewards in a controlled environment with various network failures.

Further in [16], the authors enhanced the solution of the IAPT agent by adding more exploration/exploitation actions within the range of already identified gaps from the Common Vulnerability and Exploits (CVE), expanding their repertoire of policies and rewards. The results demonstrated that incorporating solutions of the Generalized Value Iteration Pruning (GIP) type in the simulated environment can uncover multiple exploitation paths while minimizing the complexity of the policies associated with POMDP.

In references [9,17], the classic RL problem is reformulated with a focus on QL, where an off-policy temporal difference is assumed, meaning the agent estimates actions and values starting with an initial hypothesis about the environment to be exploited. In line with the findings of [17], a Capture the Flag (CTF) scenario can be envisioned, where the agent incremental learning can track seasonal and nonseasonal ports, server attacks, and the exploitation of web vulnerabilities, typically requiring between 100 to 500 iterations to achieve successful reward outcomes. Similarly, Ref. [9] concluded that work by integrating a layer known as the Double-Deep-Q-Network (DDQN), wherein the agent is able to develop more observational routes towards the attack objective, converging in fewer reward–penalty episodes.

In another context, the authors of [18] applies the concept of QL to the post-exploitation phase, where the agent learns under the premise of environments already compromised in Microsoft Windows and Linux operating systems. The QL estimation results indicate that the agent can converge towards processes for discovering plaintext credentials and privilege escalation (PEsc) with minimal policies and actions.

From the perspective of DL layers, the studies presented in [10,19] suggest that incorporating classifiers for optimal exploitation environments is a timely addition to determine whether the agent will be capable of injecting a payload into the vulnerable target. In [19], it is described that the desired characteristics originate from the types of operating systems, service versions, and exploitation modes, which—depending on the generalization of the output layer—can guide the RL agent in executing the most ideal attack during the transition of actions and policies, thereby increasing the desired reward. Similarly, Ref. [10] posits that by promoting the use of payloads from common exploitation tools such as Metasploit, SQLmap, and Weevely, the selection in the classification of test batteries can be more efficient and cost-effective in discovering optimal conditions for agent attacks.

Within simulation environments where all phases of pentesting are completed, projects such as Network Attack Simulator (NASim) [20] and PenGym [21] are noteworthy. Both projects operate within a quasi-real configuration spectrum, incorporating network environments with hosts, network topology, compromise actions, defensive devices, and vulnerable targets. NASim leverages a classic RL approach, where the agent bases its actions on a kill chain driven by satisfactory exploitation probabilities, assuming the maximum reward value for the agent. Conversely, PenGym enhances the effectiveness of the entire pentesting process through modules called ’tiny,’ where the RL algorithm focuses on session-based transition actions, achieving a faster exploratory reward value than its counterpart.

In [22], a novel architecture named Cascaded Reinforcement Learning Agents (CRLA) was introduced. This architecture addresses the challenge of discrete action spaces in an autonomous pentest simulator, where the number of actions exponentially increases with complexity across various network exploitation scenarios. It was demonstrated that CRLA identified optimal attack strategies in scenarios with large action spaces more rapidly and robustly than conventional QL agents.

Also regarding the use of BERT models in the field of cybersecurity, in [23], they proposed an approach, VE-Extractor, to extracting vulnerability events from textual descriptions in vulnerability reports, including the extraction of the vulnerability event trigger and the event arguments (such as consequence or operation), and they used a BERT QA model for this purpose.

The studies [24,25] show that Large Language Models (LLMs) offer many advantages over traditional classification methods. In [24], a BERT-based Vulnerability Detection (BBVD) method was proposed to detect software vulnerabilities from the source code level using the high-level programming language C/C++, obtaining superior results to average models based on traditional classifications. The authors of [25] presented an automated categorization of vulnerability data using DL. In this paper, they found that BERT designs fitted with LSTM outperformed standard models in precision, F1 score, accuracy, and recall, also demonstrating the advantage of using LLM models in the field of cybersecurity.

Ultimately, in reference [26], a dataset of 1813 CVEs annotated with all corresponding MITRE ATT&CK techniques was presented, and models were proposed to automatically link a CVE to one or more techniques based on the textual description of the CVE metadata. Therein, they established a robust baseline that considers classical machine learning models and state-of-the-art pre-trained BERT-based linguistic models while countering the highly imbalanced training set with data augmentation strategies based on the TextAttack framework.

## 3. Methods and Materials

**Figure 2:**
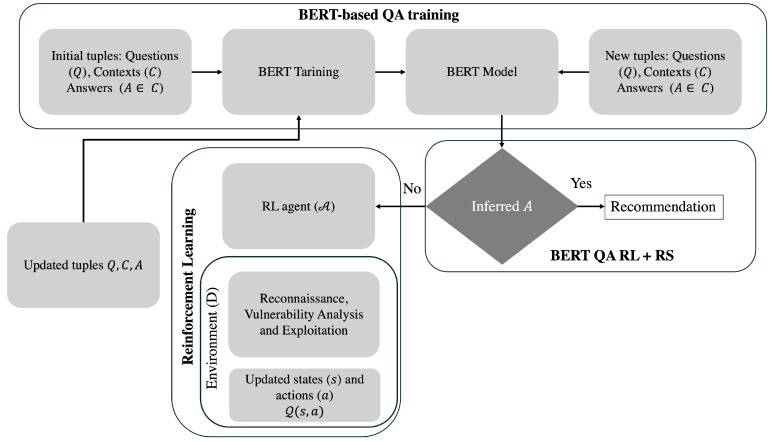
General diagram of the proposed architecture showing the interaction between the RL agent and the recommender system to obtain as output a suggestion of attacks to be performed on the available machine.

In Figure 2, the workflow of BERT QA RL + RS is illustrated.

To describe the proposal represented in Figure 2, consider a traditional scenario where a pentester follows the guidelines established by NIST SP 800-115, which define a structured methodology for conducting black-box penetration tests. Initially, as in any work with estimators during the BERT-based QA training phase, a pre-trained model is unfrozen and fine-tuned with a dataset related to the necessary QA context to recalibrate the weights and customize it for the specific task. In this case, the training involves various historical CWE cases across multiple domains, including recognition, vulnerability analysis, and exploitation. These cases are diverse and objective-driven, organized into tuples consisting of questions (*Q*) specifying objectives to test, contexts (*C*) detailing how the solution to *Q* is formulated, and A∈C, which is the contextualized answer that directly addresses *Q*.

Once the BERT QA model is generated, the pentester might begin with limited information about the target, such as IP addresses, URLs, domain names, or topological layouts. Based on this information, the pentester would determine and experiment with various tests to identify service infrastructure and preliminary vulnerabilities, assess their exploitability, and propose mitigation measures. By querying the trained BERT QA model, an inferred RS response to the question may be obtained. However, if the response is unsatisfactory or not present, the following scenario involving the Reinforcement Learning (RL) phase can be considered:

The workflow begins when the pentester defines the evaluation objectives as a query *Q*, which structures the input for the BERT QA system while aligning with the recommendation of NIST SP 800-115 to clearly establish the scope and objectives. For example,

$$\begin{array}{c} Q=What\;tests\;do\;you\;recommend\;for\;a\;Class\;C\;IP\;address,\;with\;Ubuntu\;20.0\\ operating\;system,\;running\;an\;Apache\;PHP\;5.2.4\;server? \end{array}$$

According to the guidelines of NIST SP 800-115, this query initiates the recognition phase by focusing on the target’s characteristics. If *Q* matches a context *C* already present in the knowledge base of BERT QA RL + RS, an inferred response A∈C is generated. For example,

$$\begin{array}{c} C=According\;to\;CWE\text{-}116,\;CWE\text{-}79,\;and\;CWE\text{-}94,\;with\;improper\;neutralization\\ of\;resource\;inputs\;enabling\;potential\;remote\;code\;execution,\;it\;is\;possible\;to\;use\;a\;proof\\ of\;concept\;for\;XSS\;and\;then\;inject\;arbitrary\;code\;by\;modifying\;functions.lib.php. \end{array}$$

*The response A includes actionable insights into vulnerabilities and aligns with the emphasis of NIST SP 800-115 on identifying and documenting specific risks.*

In cases where no response can be inferred, the query *Q* is transferred to the RL agent (A). Based on the decomposition of *Q*, A identifies which pentesting attributes are most suitable for reconnaissance, vulnerability analysis, and exploitation within a controlled environment D. A training process is initiated, where Q represents the matrix of states *s* and actions *a* that A must observe to determine successful interaction episodes for evaluating the desired objective. Over successive iterations, the RL agent A maximizes the effectiveness of the reward sequence across the pentesting steps. When no further paths remain to be explored, the output of A provides a new tuple (Q,C,A) to recalibrate the weights of the BERT QA model, incorporating new information for future recommendations. This iterative process ensures a dynamic and adaptive penetration testing approach.

When BERT QA RL + RS is integrated with new (Q,C,A) tuples, the pentester benefits from the combination of NIST SP 800-115 and the BERT QA RL system, resulting in potential new RS recommendations, as demonstrated in the following phases:

- *Planning and Preparation:* NIST SP 800-115 emphasizes defining clear objectives and scoping the penetration test. In this workflow, *Q* establishes these objectives, while the BERT QA RL + RS system aligns them with pre-trained contexts *C*, offering immediate actionable insights or delegating tasks to A when novel scenarios arise.
- *Recognition:* According to NIST SP 800-115, identifying active hosts, open ports, and running services is critical. The RL agent A operationalizes this by automating reconnaissance tasks using tools such as Nmap, correlating findings with known configurations in the BERT QA RL + RS system. This accelerates the discovery phase while ensuring consistency.
- *Vulnerability Identification:* NIST SP 800-115 recommends correlating reconnaissance data with known vulnerabilities. The RL agent A cross-references identified software versions and services against CWE and CVE databases, enriched by the contextual understanding provided by the BERT QA RL + RS system, to identify vulnerabilities with actionable clarity.
- *Exploitation:* NIST SP 800-115 advises conducting controlled exploitation to validate findings. Leveraging outputs from BERT QA RL + RS, the RL agent A selects optimal paths for proof-of-concept attacks using tools such as Metasploit, testing vulnerabilities like CWE-94 by attempting CI in functions.lib.php.
- *Reporting and Recommendations:* NIST SP 800-115 underscores the importance of documenting findings and proposing mitigations. Here, the tuple (Q,C,A) consolidates the test results, integrating newly discovered insights into the BERT QA RL + RS knowledge base. For example, the system may recommend upgrading PHP to version 7.4 to mitigate CWE-94 or implementing input validation to address CWE-79.

By combining the structured methodology of NIST SP 800-115 with the adaptive capabilities of the BERT QA RL + RS system, this workflow automates and optimizes key phases of penetration testing. The state–action matrix Q ensures iterative learning from A, while continuous updates to the BERT QA RL + RS system incorporate novel scenarios, enabling effective handling of diverse and previously unseen configurations.

In Section 3.1, Section 3.2 and Section 3.3, the steps of the BERT QA RL + RS strategy are described in depth.

### 3.1. BERT-Based QA Training

BERT [27] represents a series of pre-trained transformer-based models designed for various natural language understanding tasks. By utilizing a masked language model (MLM) schema, BERT enables predictions across multiple outputs, including classification, next-word and next-sentence prediction, term clustering, and inferring questions related to specific domain contexts.

In the architecture of BERT for QA, the core components consist of a query *Q*, which is framed as an argumentative formulation within a knowledge domain. For example, in the case of a RS tailored for penetration testing, *Q* could be defined as a set of features linked to target attributes, aiming to infer the optimal actionable pathway for reconnaissance, vulnerability assessment, and exploitation.

Associated with *Q* is a context, which provides detailed information on the presupposition intended for inference, serving as the referential framework toward a factual response *A*. This response *A* outlines how to conduct asset reconnaissance, identifies recognized assets that exhibit vulnerabilities, and specifies the steps to exploit these vulnerabilities. Depending on the target’s unique scenario, *A* may also include steps for post-exploitation.

The response A∈C is a specific statement delineated within the context *C* by its starting and ending indices, $A_{S}$ and $A_{E}$, as shown below:

$$\begin{array}{c} C=According\;to\;CWE\text{-}116,\;CWE\text{-}79,\;and\;CWE\text{-}94,\;with\;improper\\ neutralization\;of\;resource\;inputs,\;enabling\;potential\;remote\;code\;execution,\\ \overset{A_{S}}{\overset{︷}{i}}t\;is\;possible\;to\;use\;a\;proof\;of\;concept\;for\;XSS\;and\\ then\;inject\;arbitrary\;code\;by\;modifying\;functions.lib.ph\underset{A_{E}}{\underset{︸}{p}}. \end{array}$$

where $A_{S}=p$ indicates the starting index at position 115, and $A_{E}=p$ indicates the ending index at position 210 within the characters of *C*.

**Figure 3:**
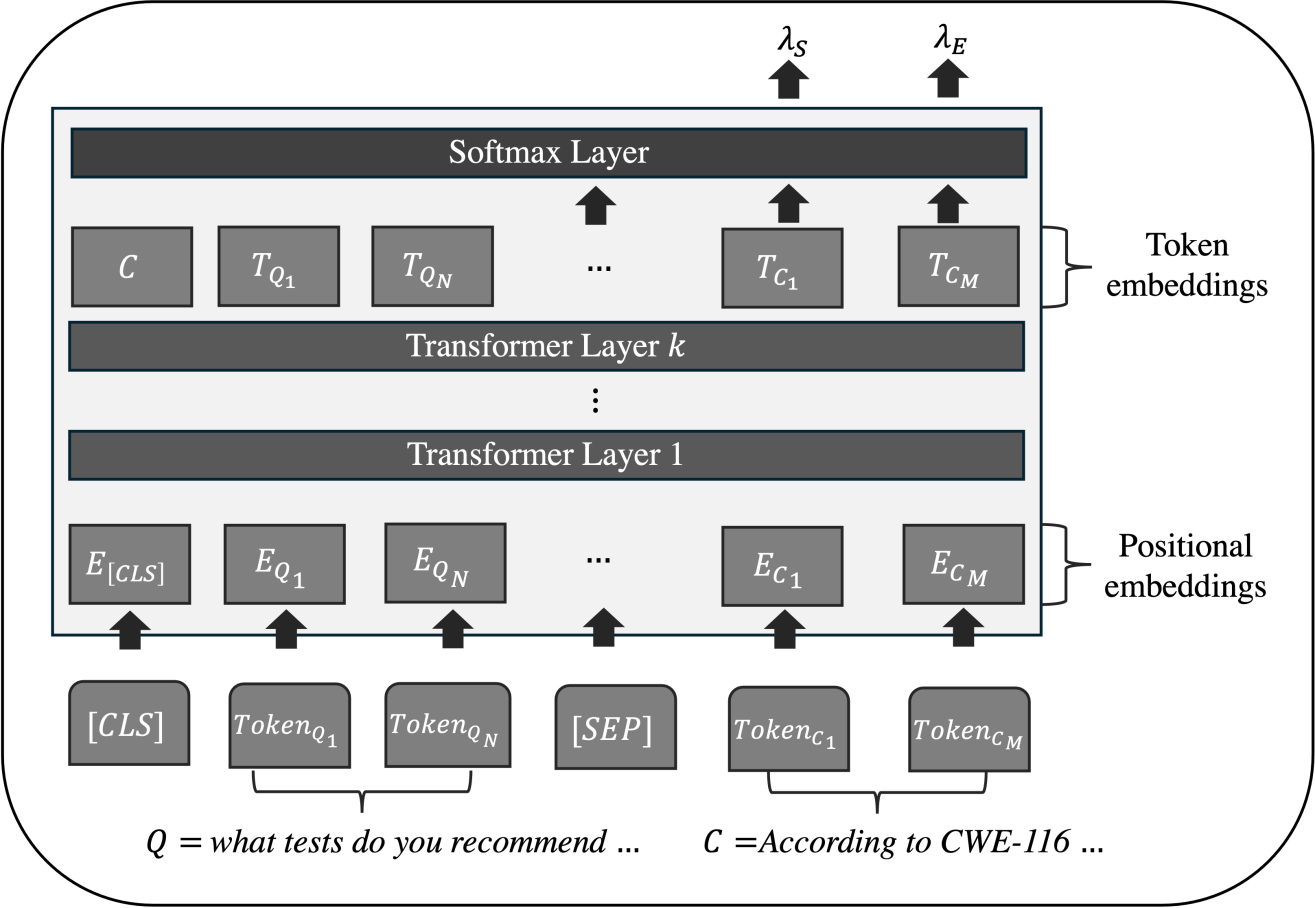
General architecture of BERT for QA. Note how *Q* and *C* are included as inputs, separated by a special token, [SEP], which indicates the boundary between the two sequences. Additionally, the [CLS] token signifies that the sequence will be used in a masked classification model, facilitating the emulation of potential answer selection.

Figure 3 schematizes the mechanism by which BERT models, utilized in QA tasks, train the sample set for RS within the context of penetration testing, thereby enabling the generation of predictions related to *A*.

The inputs for BERT in QA RS tasks are tuples Q,C,A, with A∈C, and the respective start and end indices $A_{S}$ and $A_{E}$. At this stage, the sequences *Q* and *C* must be tokenized, including start and separation tokens between *Q* and *C*, such as [CLS] and [SEP]. The [CLS] token is placed at the beginning of the sequence to indicate a classification task, while the [SEP] token separates different sentences or segments in the text, as shown in Equation (1):

(1) $$I=\left[\mathrm{CLS}\right]+{\mathrm{token}}_{Q_{1}}+\cdots +{\mathrm{token}}_{Q_{N}}+\left[\mathrm{SEP}\right]+{\mathrm{token}}_{C_{1}}+\cdots +{\mathrm{token}}_{C_{M}}$$

In this equation, *I* represents the input to the BERT QA RL + RS model. The tokens ${\mathrm{token}}_{Q_{\left\{1,\cdots ,N\right\}}}$ and ${\mathrm{token}}_{C_{\left\{1,\cdots ,M\right\}}}$ denote the *N*-th and *M*-th tokens of *Q* and *C*, respectively.

Each token ${\mathrm{token}}_{Q_{\left\{1,\cdots ,N\right\}}}$ and ${\mathrm{token}}_{C_{\left\{1,\cdots ,M\right\}}}$ is transformed into a high-dimensional embedding vector, capturing the context, order, and relationships among tokens in *I*. In BERT, two types of embeddings are calculated: the token embeddings $E({\mathrm{token}}_{Q_{\left\{1,\cdots ,N\right\}}},{\mathrm{token}}_{C_{\left\{1,\cdots ,M\right\}}})$, which capture the vocabulary-based meaning of tokens for *Q* and *C*; segment embeddings, distinguishing between tokens of *Q* and *C* and aiding in the interpretation of each sequence, as shown in Equation (2).

(2) $$S\left({\mathrm{token}}_{Q_{1}},\cdots ,{\mathrm{token}}_{Q_{N}},\;\cdots ,{\mathrm{token}}_{C_{1}},\cdots ,{\mathrm{token}}_{C_{M}}\right)\in R^{d}$$

The segment embedding vector *S* lies within the sequence dimension *d*, corresponding to the maximum length of *I*.

Positional embeddings analyze the structure and order of each ${\mathrm{token}}_{Q_{\left\{1,\cdots ,N\right\}}}$ and ${\mathrm{token}}_{C_{\left\{1,\cdots ,M\right\}}}$, enabling identification of positional context within the sequence and the relationships between *Q* and *C* to locate the potential answer *A* embedded in *C*, as defined in Equation (3).

(3) $$P\left({\mathrm{token}}_{Q_{1}},\cdots ,{\mathrm{token}}_{Q_{N}},\;\cdots ,{\mathrm{token}}_{C_{1}},\cdots ,{\mathrm{token}}_{C_{M}}\right)\in R^{d}$$

The positional embedding vector *P* also lies within the sequence dimension *d*, corresponding to the maximum length of *I*.

The concatenated representation of tokens, defined by Equation 4, combines the embeddings as follows:

(4) $$H_{\left\{{\mathrm{token}}_{Q_{1}},\cdots ,{\mathrm{token}}_{Q_{N}},\;\cdots ,{\mathrm{token}}_{C_{1}},\cdots ,{\mathrm{token}}_{C_{M}}\right\}}=\left(E+S+P\right)\in R^{(n+2)\times d}$$

This combined embedding, $H_{\left\{{\mathrm{token}}_{Q_{1}},\cdots ,{\mathrm{token}}_{Q_{N}},\;\cdots ,{\mathrm{token}}_{C_{1}},\cdots ,{\mathrm{token}}_{C_{M}}\right\}}$, represents the concatenation of tokens, with (n+2)∈(N+M) denoting the total number of tokens generated by *E*, *S*, and *P*, including the special tokens [CLS] and [SEP].

The embeddings *H* then undergo *k* layers of transformation, each consisting of several interconnected elements, including Multi-Head Attention, stabilization via Normalization, a nonlinear Feed-Forward Network (FNN), and an output activation to generate the probabilistic predictions for the potential output indices of A∈C, referred to as the start logits ($\lambda_{S}$) and end logits ($\lambda_{E}$).

The Multi-Head Attention mechanism allows for simultaneous attention to different parts of the discourse between *H* of *Q* and *C*, facilitating the learning of relationships between words, both nearby and distant, within a single context. This enhances language comprehension by utilizing the pre-existing parameters *W* in the BERT model. For each attention head, three matrices are computed: The query matrix $q=H\;\cdot \;W^{q}$, which transforms *H* into a query space that establishes relationships and similarities among all tokens embedded in the sequence, allowing focused attention on each token. The key matrix $K=H\;\cdot \;W^{K}$, representing the contextual characteristics of the tokens in *H* in relation to the query matrix. The value matrix $V=H\;\cdot \;W^{V}$, which contains the information transferred between tokens based on the keys, capturing relevance, context, and the relationships learned with other tokens.

In this case, the attention in each head is calculated with $\left(q,K,V\right)\in R^{d\times d_{k}}$, where $d_{k}$ is the dimension of the *q* and *K* vectors, as shown in Equation (5):

(5) $$\mathrm{Attention}\left(q,K,V\right)=\mathrm{softmax}\left(\frac{qK^{T}}{\sqrt{d_{k}}}\right)V$$

Here, $\sqrt{d_{k}}$ acts as a normalization factor, preventing the attention scores from becoming excessively large and thus stabilizing the learning process.

When using all attention heads, the complete calculation is defined as shown in Equation (6):

(6) $$\mathrm{MultiHead}\left(Q,K,V\right)=\mathrm{Concat}\left({\mathrm{head}}_{1},\cdots ,{\mathrm{head}}_{h}\right)W^{O}$$

where ${\mathrm{head}}_{h}$ denotes the output of the h-th attention head, and $W^{O}\in R^{(h\;\cdot \;d_{k})\times d}$ is the parameter matrix used to project the concatenated output into a final dimension of *d*.

After the MultiHead attention process, the sequential output $x_{\mathrm{MultiHead}}$ passes through a feed-forward neural network (FNN) that applies nonlinear transformations to its representations. This process captures the contextual importance and unique characteristics of each element in the sequence, creating a latent language representation that integrates the information from *Q*, *C*, and the associations of *A*, as shown in Equation (7).

(7) $$x_{\mathrm{FFN}}=FNN\left(\max\left(0,x_{\mathrm{MultiHead}}\;\cdot \;W_{1}+\theta_{1}\right)\;\cdot \;W_{2}+\theta_{2}\right)$$

In this formulation, $x_{\mathrm{FFN}}$ is the FNN output; $W_{1}$ and $W_{2}$ represent BERT parameters to be adjusted for the latent language representation, and $\theta_{1}$ and $\theta_{2}$ serve as bias terms that help maintain the contextual relationships within the vocabulary range of the sequences in $x_{\mathrm{MultiHead}}$.

Consequently, normalization stabilizes learning and enhances both generalization and convergence by keeping the internal values resulting from the MultiHead and FNN layers within an appropriate range. This prevents issues of instability or gradient vanishing during training, where Norm(H+sublayer(X)) is applied. Here, *H* represents the embedding input, and *X* can refer to the output $x_{\mathrm{MultiHead}}$ or $x_{\mathrm{FFN}}$.

The results of the final operation generate an output layer where logits are calculated, representing the unnormalized scores used to estimate the probabilities for the different indices corresponding to the correct answer *A*. Let Norm be the final step following the FFN, and let the output $T=\{t_{{S|E}_{1}},\cdots ,t_{{S|E}_{n}}\}$ represent a series of token distributions over start and end positions in *T*, with weights *W* calibrated to produce an output based on the probability of all possible start and end indices for *A*, referred to as the start logits $\lambda_{S_{n}}=t_{S_{n}}\;\cdot \;W$ and end logits $\lambda_{E_{n}}=t_{E_{n}}\;\cdot \;W$.

To transform the logits into numerical indices, a softmax function must be applied. The argmax denotes the highest probability value corresponding to the predictions of *A*, as given by ${\hat{A}}_{S_{n}}=\mathrm{argmax}\left(\mathrm{softmax}\left(\lambda_{S_{n}}\right)\right)$ and ${\hat{A}}_{E_{n}}=\mathrm{argmax}\left(\mathrm{softmax}\left(\lambda_{E_{n}}\right)\right)$, where ${\hat{A}}_{S}$ is the predicted start index, and ${\hat{A}}_{E}$ is the predicted end index.

It is challenging to address all possible Q,C,A values that currently exist, including legacy, temporary, current, or zero-day vulnerabilities. However, the NIST vulnerability repository [28] provides a substantial list of approximately 93,000 records of security breaches reported by various vendors since 2013. These breaches are classified by impact level—informative, low, medium, or high—based on temporal, environmental, network, and exploit complexity factors. Following this line of reasoning, the samples from [28] include a unique submission identifier, year, characteristics, CVE (if applicable), vendor, vulnerability type, CWE, affected versions, vulnerability description, and proof of concept (if applicable).

Of the total 93,000, more than 171 distinct CWE types were captured, spread across 43,080 well-identified vulnerabilities with proofs of concept. These encompass 40,554 vendors, languages, services, and products, primarily operating systems such as Ubuntu-Linux, Fedora-Linux, Android-Linux, Windows 7, 8, 10, and Windows Server 2008, as well as Java Struts, PHP, Apache2, Nginx, C/C++, Python Flask, and OpenSuse, to generate complete tuples Q,CA. Of the remaining 41,679, only 1 or 2 of $V_{R}$, $V_{I}$, or $V_{E}$ were available, and 8421 were candidates for submission to the RL estimator.

The questions *A* will be formulated in a closed and argumentative form, containing characteristics such as the environment, possible versions, ports, and information related to the target’s surrounding space to be evaluated. On the other hand, the contextual definition of *C* will be associated with the vendor, one or more CVEs, the vulnerability, affected versions, one or more CWEs, vulnerability description, and proof of concept. Conversely, A∈C will only contain proof of concept and the indices $A_{S}$, $A_{E}$ to denote its boundary within *C*. If none exists, then *A* will be completed with a note indicating it will be sent to the RL agent for evaluation.

### 3.2. Reinforcement Learning

To formulate the RL problem in an ideal pentesting scenario, consider an agent A interacting with an environment D consisting of a set of technologies with vulnerable services V. For A to conduct pentesting tests over the scope of V, a state space $S_{t}$ is required to represent the possible configurations of the environment based on V, along with an action space $S_{a}$ that includes recognition ($V_{R}$), vulnerability identification ($V_{I}$), and exploitation ($V_{E}$) tests. Consequently, at each step, A transitions within D by selecting an action $a\in S_{a}$ given a state $s\in S_{t}$, with the goal of maximizing a reward $r:S_{t}\times S_{a}\to R$ through effective recognition, identification, or exploitation of V.

However, the previous formulation from a classical RL perspective can present challenges in highly dynamic environments, such as penetration testing scenarios, where incremental changes in technologies, services, and potential vulnerabilities—among other factors—are common. In such contexts, freedom of interaction with limited prior knowledge of the environment is essential. For this reason, Q-Learning [29] was chosen as the learning architecture, a type of RL that provides flexibility to handle dynamism and offers high convergence capabilities.

According to [13,29], Q-learning, also known as quality learning, emerges as an alternative for infinite horizon environments. This method is ideal for an agent A to navigate environments with limited knowledge, adapting its decisions as it observes environmental conditions. In this context, A learns from its actions $S_{a}$ to maximize the set of rewards $R\in R^{1\times n}$, without being subject to specific adjustment policies $\phi_{\epsilon }$.

Within penetration testing, this approach is formulated using a two-dimensional state–action matrix Q(s,a) for each pair {s,a}, where *s* denotes the state and *a* the associated action. The value of Q estimates the optimal reward r∈R obtained by performing an action *a* linked to $V_{R}$, $V_{I}$, and $V_{E}$ conditioned on s∣D. The goal is for A, with preliminary knowledge of the testing scenario in a given environment D, to accumulate the highest Q values, representing effective interactions to approximate V. Equation (8) shows the calculation of these Q values.

(8) $$Q\left(s,a\right)=\left(1-\alpha \right)\;\cdot \;Q\left(s,a\right)+\alpha \;\cdot \;[r+\gamma \;\cdot \;\max_{a^{\prime }}Q\left(s^{\prime },a^{\prime }\right)]$$

Therein, we have the following:

1. Q(s,a) represents the Q-value of the current state–action pair. If A is in an initial state s∣D with preliminary knowledge of the environment, *s* remains unknown, and A would be in the recognition stage, performing the action *a* of port or service scanning.
2. α is the learning rate, which regulates the influence of new information on updates. A high α implies that this information significantly impacts the value adjustments during the interaction of *a* within state *s*, and determining the amount of data *a* requires to recognize technologies associated with a specific port or service.
3. *r* is the reward obtained by performing action *a* in state *s*. For instance, A could receive a high reward for successfully identifying the versions of technologies linked to a particular port or service.
4. γ is the discount factor for future rewards. If A fails to recognize the port in s∣D, γ governs the importance of future versus immediate rewards, prompting A to adopt more aggressive strategies to gather information about the port and assess its potential vulnerabilities.
5. $s^{\prime }$ is the resulting state after taking action *a*. Once the services and technologies associated with a port are identified, A advances towards V in a new state $s^{\prime }$, where the action $a^{\prime }$ will focus on analyzing potential vulnerabilities.
6. $\max_{a^{\prime }}Q\left(s^{\prime },a^{\prime }\right)$ represents the maximum Q-value across all possible actions in the subsequent state $s^{\prime }$. This value corresponds to the highest expected reward that A can achieve from $s^{\prime }$ by selecting the optimal action $a^{\prime }$. In this sequence of actions, it would indicate that A has accumulated sufficient information to progress from port identification to service association and, ultimately, to vulnerability exploitation.

For the construction of the environment D, the renowned OpenAI Gym library [30], developed in version 3.x of the Python programming language, was employed. This library provides essential tools for establishing a Q-learning type reinforcement learning environment. Consequently, A was configured to train in the action space $S_{a}$ within an infrastructure consisting of two virtual machines (VMs), each with its own state space $S_{t}$ and different configurations for V.

Following the recommendations of the Cybersecurity and Infrastructure Security Agency (CISA) [31], various maturity reports were considered to determine the most suitable and reproducible scenarios in D. This approach allowed the machines to be populated with various security breaches based on the most persistent vulnerabilities that, according to the CISA Advisory AA23-215, continue to impact production systems.

Although the environments D in this RL scenario are intentionally vulnerable, their configuration reflects common and critical real-world security problems, mimicking possible attack schemes. However, in most RL problems, observations may be unpredictable, as in production systems, since the horizon over {s,a} can increase over time.

To replicate back-end production conditions, the reported scenarios are structured to showcase an incremental progression, starting with network services, transitioning to web vulnerabilities, and culminating in exploitation through misconfigurations. This approach ensures the scenarios are both comprehensive and interconnected, demonstrating realistic attack pathways:

- *Network Services:* The initial setup includes vulnerabilities in services such as FTP, SSH, and Telnet. For example, a misconfigured FTP service exposes sensitive directories, enabling unauthorized access to confidential files. This stage establishes a foundational exploitation route in network services.
- *Web Vulnerabilities:* Building upon the network exploitation, the progression incorporates web-based weaknesses such as XSS and SQL-injection (SQLi). For instance, leveraging credentials from the compromised FTP server, an attacker could exploit an insecure web application to inject malicious SQL queries, gaining unauthorized database access.
- *Misconfiguration Scenarios:* The final stage addresses critical configuration failures. Expanding on the previous exploit, a poorly configured database instance with no password protection allows for further data extraction. This demonstrates the compounding effect of misconfigurations as a vulnerability multiplier.

According to what has been expressed previously, approximately 1520 vulnerable configurations were assembled for the virtual machines (VMs) in the environments D. While it is infeasible to cover all existing vulnerabilities due to their dynamic and evolving nature, these configurations focus on the most common and impactful vulnerabilities observed in real-world scenarios. This ensures a practical and representative framework for evaluating penetration testing architectures, emphasizing network services, web vulnerabilities, and system misconfigurations to simulate realistic attack pathways.

The detailed configurations of the VMs supporting these scenarios for the environments D are as follows:

- *First Machine:* This machine contains a series of configurations $S_{t}$ based on the Linux 20.04 operating system, built upon the Metasploitable 2 [32] framework, with a set of intentionally vulnerable underlying services that facilitate practice in command injection (CI); misconfigurations; brute-force attacks (BFAs); exposed directories (EDs); outdated components (OCs); and failures in cryptographic flaws (CFs), authentication bypass (AB), and integrity, enabling various penetration testing exercises. The services include vulnerable versions of File Transfer Protocol (FTP), Secure Shell (SSH), Telnet, Tomcat, Network File System (NFS), UnrealIRCd, and Apache within the scope of network services; database management systems with security flaws in MySQL and PostgreSQL; a minimal version of Damn Vulnerable Web Application (DVWA) [33], which presents vulnerabilities such as XSS, directory traversal (DT), insecure deserialization (ID), arbitrary CI (ACI), and PEsc; file-sharing services through Samba; and RPC services—specifically Distcc and RExec—both configured in ways that allow vulnerability exploitation. In total, this machine offers more than 100 exploitation paths, 50 CVEs with proof-of-concept exploits, and over 40 reported weaknesses in the CWE (Common Weakness Enumeration) [34].
- *Second Machine:* Similarly configured with Ubuntu 20.04, this machine hosts a series of $S_{t}$, including Metasploitable 3, an updated version of its predecessor featuring vulnerabilities in Windows 8 and 10 operating systems specifically targeting services such as Tomcat, Python’s Flask, and Jenkins. Additionally, it offers several exploitation paths for the new version of Microsoft RDP. On the other hand, it enables handling PoCs for Fedora with vulnerable applications such as PHP, Apache Struts for Java, FTP, and Webmin. In total, it allows for the analysis of patterns for RCE, XSS, DT, information leakage, insecure configuration (IC), ID, API abuse (AA), CF, AB, and authorization flaws (AFs). Overall, there are 80 paths for reconnaissance, vulnerability analysis, and exploitation, with 40 identified CVEs and over 50 CWEs.

When D is in its initial state and the series of transitions between the different configurations of s∣D with various presentations of V begins, A is preconfigured with three sensors aligned to the pairs {s,a} at any given moment. For the recognition phase, Network Mapper (Nmap) [35] was employed, a well-known analyzer of technologies, services, and protocols associated with network ports using both passive and aggressive scanning techniques. In the vulnerability analysis states, an extension called Nmap Vulners [36] was used, which compares the data collected in the recognition phase with previously reported V patterns in the CVE database. When *a* is in the exploitation block, it directs operations toward various Metasploit [37] modules, a suite that integrates confirmed V payloads, to establish the target, attack path, and exploitation method.

To ensure gradual and continuous learning, an exploration-exploitation strategy was implemented as part of the Q-Learning algorithm, which operates iteratively, progressively refining the estimates of the Q(s,a) values. Over multiple episodes of interaction with D, A adjusts its policies $\phi_{\epsilon }$, when necessary, to maximize the cumulative rewards *r* of the state–action process (s,a) [38], as detailed in Algorithm 1.

Each successful *a* yields a specific reward, ranging from {1,3}, depending on the complexity of the attack, with a maximum cumulative reward of 3: 1 for $V_{R}$ stage, 2 for $V_{I}$, and 3 for $V_{E}$. In the event of failed attempts, γ adjusts to enable A to return to the exploration–exploitation process at the current state *s* but with improved control over the search path through a more flexible $\phi_{\epsilon }$ while recalculating ϵ to optimize the desired path until it converges at $Q(s^{\prime },a^{\prime })$.

The RL agent does not rely on any preexisting dataset for training. Instead, it learns through direct interaction with a simulated environment D, which includes the two intentionally vulnerable virtual machines (VMs) described above. The agent uses trial-and-error exploration to improve its policy, receiving rewards based on the success of its actions. This design ensures that the training process adapts to dynamic scenarios rather than static data, mitigating risks of overfitting. Furthermore, the VM configurations were periodically altered to introduce variability, enhancing the generalization capabilities of the RL agent.
**Algorithm 1** Q-Learning for $V_{R}$, $V_{I}$, and $V_{E}$ tests.  1:**Initialization:**  2:Initialize the Q-table Q(s,a) with zeros for all state-action pairs (s,a)  3:Set the learning rate α, discount factor γ, and the parameter ϵ for the policy $\phi_{\epsilon }$  4:**for** each episode **do**  5:    Initialize the state *s* with the initial configuration of D. In the initial state *s*, A performs a reconnaissance process of all available virtual machines  6:    **while** the state *s* is not terminal **do**  7:        Select the action *a* based on the policy $\phi_{\epsilon }$  8:        Execute action *a*, observe reward *r* and the new state $s^{\prime }$  9:        **if** *a* pertains to $V_{R}$ **then**10:           Perform reconnaissance using Nmap11:        **else if** *a* pertains to Vulnerability $V_{I}$ **then**12:           Conduct vulnerability identification using Nmap Vulners13:        **else if** *a* pertains to $V_{E}$ **then**14:           Conduct exploitation using Metasploit15:        **end if**16:        Select $a^{\prime }$ as the action that maximizes $Q(s^{\prime },a^{\prime })$17:        **Update Q(s,a) using the Bellman equation:**
$$\;Q\left(s,a\right)\leftarrow Q\left(s,a\right)+\alpha \left[r+\gamma Q\left(s^{\prime },a^{\prime }\right)-Q\left(s,a\right)\right]$$18:        Update the state $s\leftarrow s^{\prime }$19:    **end while**20:**end for**

The iteration episodes for A conclude under two conditions: the first is the successful completion of $\forall \{V_{R},V_{I},V_{E}\}$, and the second is truncation, which occurs if the agent cannot complete any of the specified actions toward $Q(s^{\prime },a^{\prime })$.

This strategy defines a parameter ϵ to guide A in selecting effective *a* for each *s*. At each stage, A evaluates the probability of exploring a new action or leveraging a known one. If this probability exceeds ϵ, A selects the most effective $a|a^{\prime }$ learned so far. If lower, A selects a random $a\in S_{a}$, ensuring broad exploration of the action space. As training advances, exploration gradually decreases, enabling A to converge toward optimal $Q(s^{\prime },a^{\prime })$. A decay rate $\epsilon_{decay}$ is applied to ϵ, steadily reducing the probability of random selection until it reaches zero, at which point A will consistently execute only the most effective actions.

After executing A and completing the Q(s,a) table, the pairs (s,a) with the highest rewards r∈R over multiple episodes are compiled into a JSON interaction dataset. The output will consist of four keys: target to evaluate, contextual characteristics of the target, parameters used for $V_{R}$, identified vulnerabilities in $V_{I}$, and steps for successful exploitation of $V_{E}$. Some rows will contain complete data for all three stages—reconnaissance, vulnerability identification, and exploitation—while others may contain only the first two stages or just the initial stage. In cases where A did not reach its goal, these columns will be marked as failed attempts, which will serve the RS in determining that, according to a given *Q*, there is no *A* capable of responding to the query.

**Figure 4:**
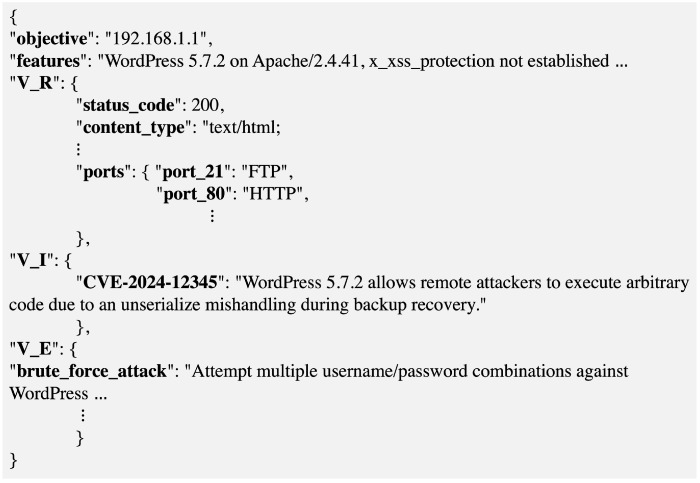
The JSON format begins with an objective key to define the target, followed by essential characteristics in the features key, required to initialize the RL estimator. When A completes its training, it outputs reconnaissance results $V_{R}$ in the V_R key, identifies of one or more vulnerabilities $V_{I}$ in the V_I key, and outputs from the exploitation episode $V_{E}$ in the V_E key, which serve as input for the BERT QA RL + RS.

Once A completes its exploration/exploitation in D, all JSON objects are consolidated into a single dataset for subsequent ingestion by the BERT QA RL + RS. Figure 4 provides an example of a JSON object generated as a result of the RL process.

### 3.3. BERT QA RL + RS

In order to integrate BERT QA RL + RS into the RL estimator, and under the assumption that a question *Q* does not yield a satisfactory answer $\hat{A}$, it is essential to define a transition function, as outlined in [39]. Initially, it is assumed that the RL process starts with an empty table Q(s,a) in its first iteration.

Let δ be a confidence threshold based on a specific performance metric for BERT QA RL + RS. If the predicted answer $\hat{A}\in \left\{{\hat{A}}_{S},{\hat{A}}_{E}\right\}$ has a probability $P(\hat{A}|Q,C)<\delta$ of adequately addressing the question *Q* in the context *C*, then the RL estimator, using the mapping M:(Q,C)→D, will seek the optimal environment D to initialize the state–action pairs in the table $Q{\left(s,a\right)}_{\left\{Q,C\right\}}$.

In this scenario, A starts in an initial state $s_{0}$ and performs an action $a_{0}$. After the first iteration, M re-evaluates (Q,C) to confirm or adjust D, allowing the RL estimator to proceed to the next iteration $\left(s_{i+1},a_{i+1}\right)$ until it converges at $\left(s^{\prime },a^{\prime }\right)$. If convergence is not achieved—that is, if there are no values within the spaces $S_{t}$ and $S_{A}$ that yield a new *A*—a context *C* and an answer *A* will be returned, indicating that there are no routes for question *Q* in any of the spaces $V_{R}$, $V_{I}$, and $V_{E}$.

Conversely, if a transition to $V_{R}$, $V_{I}$, or $V_{E}$ is feasible, then the output keys from the RL estimator will be concatenated within context *C*, with $V_{R}$ assigned to the V_R key, $V_{I}$ to V_I, and $V_{E}$ to V_E, forming a new tuple (Q,C,A), which will be added to the BERT QA RL + RS model.

The Bellman equation for updating the value of Q(s,a) for a new question *Q* with an uninferred answer $\hat{A}$ is expressed in Equation (9):

(9) $$Q{\left(s_{\left\{1,\cdots ,u\right\}},a_{\left\{1,\cdots ,u\right\}}\right)}_{\left\{Q_{\left\{1,\cdots ,u\right\}},C_{\left\{1,\cdots ,u\right\}}\right\}}=\left(1-\alpha \right)\;\cdot \;Q\left(s,a\right)+\alpha \;\cdot \;\left[r+\gamma \;\cdot \;\max_{a^{\prime }}Q\left(s^{\prime },a^{\prime }\right)\right],$$

where $Q{\left(s_{\left\{1,\cdots ,u\right\}},a_{\left\{1,\cdots ,u\right\}}\right)}_{\left\{Q_{\left\{1,\cdots ,u\right\}},C_{\left\{1,\cdots ,u\right\}}\right\}}$ represents the updated value for contexts *Q* and *C* in the *u*-th iteration of an uninferred $\hat{A}$, α is the learning rate, *r* is the reward obtained, and γ is the discount factor that values future rewards. This iterative process continues until each new answer $\hat{A}$ reaches an acceptable confidence level or until the RL estimator A completes its training in environment D, as defined by M.

Since the weights of the BERT model are frozen after the last training process, it is necessary to incorporate the new inputs $Q_{u}$, $C_{u}$, and $A_{u}$. As discussed in Section 1, new inputs $I_{u}$ are generated to construct a *u*-th version of the tokens:

$$I=\left[\mathrm{CLS}\right]+{\mathrm{token}}_{Q_{N_{u}}}+\cdots +\left[\mathrm{SEP}\right]+\cdots +{\mathrm{token}}_{C_{M_{u}}}$$

This yields a new representation with segment embeddings $S_{u}$, positional embeddings $P_{u}$, and a final concatenated representation *H* now using the latest BERT parameters *W*, as shown in Equation (10).

(10) $$H=BERT(Q_{u},C_{u},W)$$

In this equation, BERT represents the latest trained model, $Q_{u}$ and $C_{u}$ refer to the new question and context for the *u*-th input, and *W* denotes the updated model weights.

As a next step, the weights *W* can be unfrozen for the *u*-th inputs and recalibrated through a fine-tuning layer that utilizes the new representations *H* and the answers $A_{u}$, with their start and end indices, $\left\{A_{S_{u}},A_{E_{u}}\right\}$, as specified in Equation (11).

(11) $${\hat{A}}_{u}=\sigma \left(W^{T}H+W_{0}\right)$$

Here, $W^{T}$ represents the weights to be recalibrated based on the new prediction $A_{u}$, σ is the softmax function, and $W_{0}$ serves as the bias term. To complete the calibration of the weights *W* with respect to the new answers $A_{u}$, Equation (11) is decomposed into the new logits distributions for the start $\left(\lambda_{S_{u}}\right)$ and end $\left(\lambda_{E_{u}}\right)$ indices, generating a cross-entropy loss function L, as expressed in Equation (12).

(12) $$L\left(W_{A_{S_{u}}},W_{A_{E_{u}}}\right)=-\log\left({\hat{A}}_{S_{u}}\right)-\log\left({\hat{A}}_{E_{u}}\right)$$

In this context, $W_{A_{S_{u}}}$ and $W_{A_{E_{u}}}$ denote the decomposed weights for the start and end indices. The normalized predictions of these indices are calculated as

$${\hat{A}}_{S_{u}}=\mathrm{argmax}\left(\mathrm{softmax}\left(\lambda_{S_{u}}\right)\right)\;\mathrm{and}\;{\hat{A}}_{E_{u}}=\mathrm{argmax}\left(\mathrm{softmax}\left(\lambda_{E_{u}}\right)\right)$$

The weights $\left\{W_{A_{S_{u}}},W_{A_{E_{u}}}\in W\right\}$ and the bias $W_{0}$ will be iteratively readjusted as new *u* tuples are ingested from the RL estimator, optimizing their recalibration as outlined in Equations (13) and (14).

(13) $$W:=W-\eta \nabla_{W}L\left(W\right)$$

(14) $$W_{0}:=W_{0}-\eta \nabla_{W_{0}}L\left(W_{0}\right)$$

where η represents the learning rate, and ∇ denotes the gradient change applied to update the BERT weights as new values of $Q_{u}$, $C_{u}$, and $A_{u}$ are added.

Ultimately, while the reliance on static datasets such as CVE and CWE serves as a foundation, the system’s iterative interaction between the RL estimator and the dynamically updated contextual knowledge ensures adaptability. This process leverages new inputs $Q_{u}$, $C_{u}$, and $A_{u}$, as described, to enhance the model’s ability to address novel attack vectors. By refining the weights *W* and recalibrating the fine-tuning layer for each new iteration, the system mitigates limitations associated with static data reliance, ensuring that it can adapt to evolving security landscapes. Such adaptability aligns with best practices for reinforcement learning systems, as outlined in [40], enabling continuous learning and improved response generation.

## 4. Results

In this study, the results are systematically divided into three subsections to emphasize the distinct contributions of the proposed BERT QA RL + RS framework. This structure facilitates a detailed analysis of each component, highlighting their respective contributions and performance. The subsections and principal findings are outlined as follows:

- *Section 4.1 Computational Efficiency of the Reinforcement Learning Agent:* This subsection evaluated the RL agent’s learning behavior, convergence, and computational efficiency across 16 hyperparameter configurations. Configuration 12 emerged as the most optimal, achieving the highest cumulative rewards and the shortest episode lengths. These findings highlight the agent’s ability to effectively balance exploration and exploitation within the state-action space, as well as its robustness under varying initial conditions
- *Section 4.2 Performance Analysis of BERT QA Models:* The study compared the computational efficiency and QA accuracy of three BERT-based models: BERT, RoBERTa, and DistilBERT. While DistilBERT demonstrated superior computational efficiency, requiring less training time and resources, RoBERTa achieved the highest QA accuracy with an F1-Score of 99.99%. These results emphasize the trade-offs between computational resource demands and QA precision, enabling informed decisions about model selection based on specific application needs.
- *Section 4.3 Combined RL and BERT QA RL + RS Framework:* The integration of the RL agent with BERT QA RL + RS demonstrated its practical utility in prioritizing critical vulnerabilities within an automated penetration testing environment. The system effectively prioritized the most important vulnerabilities, with 14 out of 23 recommendations aligning with the top vulnerabilities in the CVE dataset. Additionally, the total training time for the integrated framework was approximately 1129.4 min, and the average task execution time was 23 min, which included RL decision-making and BERT inference. These results underscore the practical applicability of the integrated framework in prioritizing and addressing high-risk vulnerabilities in real-world scenarios.

### 4.1. Computational Efficiency of the Reinforcement Learning Agent

Assuming that a pentester intends to consult the process of a test battery with a *Q*, there are two possible paths: BERT QA RL + RS can infer an $\hat{A}$, or it can submit it to the agent A to recalibrate BERT QA RL + RS through the exploration and exploitation of $V_{R}$ and $V_{I}$ for new tuples Q,C,A. Taking this last hypothesis into account, the NIST dataset, already structured in tuples $Q,C,A,A_{S},A_{E}$, was divided into 80% for training and 20% for testing without replacement. First, the RL estimator was evaluated, assuming that there are $\hat{A}$ instances not inferred in BERT QA RL + RS. The following performance metrics are presented in this context:

- *Cumulative Reward (CR):* The sum of rewards obtained by the agent over an episode or a period. The cumulative reward equation evaluates the total rewards accumulated by the agent, with the objective of maximizing this sum, as formulated in Equation (15):
  (15) $$R_{i}=\sum_{j=i}^{T}r_{j}$$
  where $R_{i}$ is the cumulative reward at instant *i*, *T* is the time horizon, and $r_{j}$ is the reward at instant *j*.
- *Episode Length (EL):* Represents the number of steps (actions) taken by the agent to complete an episode, as determined by Equation (16).
  (16) $$L_{i}=\sum_{j=1}^{N}\Delta_{j}$$
  Here, $L_{i}$ denotes the episode length at instant *i*, *N* is the total number of steps, and $\Delta_{j}$ is the duration of each step *j*. This metric evaluates the total number of actions required by the agent to complete its task.
- *Policy Entropy (PE):* Measures the uncertainty of the agent’s policy, which is useful for evaluating its level of exploration. Policy entropy is defined in Equation (17):
  (17) $$EP\left(P\right)=-\sum_{a}P\left(a\right)\;\cdot \;\log\left(P\left(a\right)\right)$$
  where P(a) is the probability of taking action *a*. A high entropy value indicates greater exploration in the selection of actions, while low values indicate a more stable and defined policy.
- *Mean Squared Error (MSE):* Evaluates the accuracy of the agent’s predictions compared to real values in the environment, as shown in Equation (18):
  (18) $$\mathrm{MSE}=\frac{1}{N}\sum_{i=1}^{N}{\left(Q\left({\hat{A}}_{i}\right)-Q\left(A_{i}\right)\right)}^{2}$$
  where *N* is the number of examples, $Q({\hat{A}}_{i})$ represents the agent’s predictions, and $Q(A_{i})$ represents the real values in the *i*-th iteration. This mean squared error quantifies the difference between the actions predicted by the agent and those observed, providing a measure of accuracy.

On the other hand, Table 1 presents the parameters used for the RL estimator, specifically for A. Each parameter distinctly influences the iterative behavior, with values (Value 1, Value 2) that, according to [41], have been shown to be effective across the desired *i* episodes for $V_{R}$, $V_{I}$, and $V_{E}$.

As suggested in [42], simulations are particularly valuable in tasks where real-world interactions are costly or infeasible, enabling agents to learn robust behaviors in a risk-free environment. This principle emphasizes the necessity of conducting multiple simulations to validate the consistency and robustness of the RL agent. Through these simulations, it is possible to ensure that the results are reproducible under identical conditions, confirming that outcomes are not merely coincidental or influenced by stochastic external factors but reflect the expected performance of the agent.

To verify the consistency of the agent A, two simulations were carried out under identical initial conditions. The first simulation (*Sim. 1*) established a controlled environment D with predefined states $S_{t}$, actions $S_{a}$, and a fixed reward structure. The primary objective of *Sim. 1* was to iteratively optimize Q(s,a), allowing A to adaptively learn an optimal policy. The second simulation (*Sim. 2*) replicated these conditions to confirm that the policies learned in *Sim. 1* were not influenced by stochastic factors such as random initialization or environmental noise. Any discrepancies between the results of *Sim. 1* and *Sim. 2* would indicate potential sensitivity issues, as noted in [40], where reproducibility in RL often depends on addressing randomness in exploration strategies and environmental dynamics.

The iterative learning process ensures that A incrementally improves its policy $\phi_{\epsilon }$, maximizing the cumulative rewards r∈R over multiple episodes. During training, the progressive reduction of ϵ decreases random exploration, focusing A on exploiting the best-known actions for each state. The consistency between *Sim. 1* and *Sim. 2* validates the reliability of Q updates and the adaptability of A to dynamic environments.

**Figure 5:**
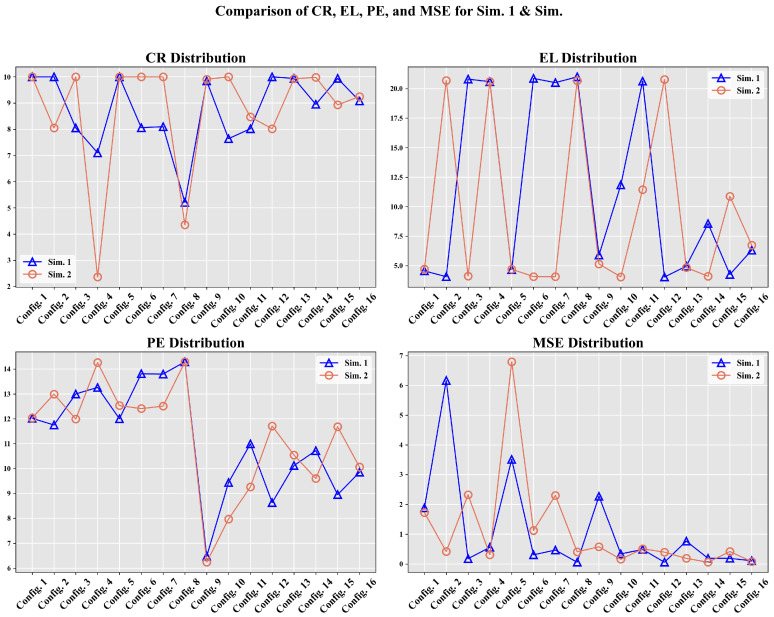
Evolution of the metrics MSE, CR, EL, and PE across 16 configurations for Sim. 1 and Sim. 2 RL simulations.

The results obtained with the experimental design are presented below, analyzing the influence of the variation in selected hyperparameters. Table 2 shows the configurations used for A execution based on combinations of hyperparameters. Consequently, Figure 5 shows the evolution of MSE, CR, EL, and PE across 16 configurations for *Sim. 1* and *Sim. 2* in two RL simulations.

Taking Figure 5 as a reference, where the MSE metrics for the two simulations (Sim. 1 and Sim. 2) are compared, a quantitative hypothesis was also proposed to evaluate overfitting. Overfitting in RL environments [43] occurs when A adjusts its policy excessively to a specific configuration or initial seed, thereby losing its capacity for generalization under slightly different conditions.

To quantify the stability of the behavior of A between simulations, the normalized relative difference of the MSE was defined, as shown in Equation (19).

(19) $$\delta_{i}=\frac{\left|{\mathrm{MSE}}_{\mathrm{Sim}1,i}-{\mathrm{MSE}}_{\mathrm{Sim}2,i}\right|}{{\mathrm{MSE}}_{\mathrm{Sim}1,i}+{\mathrm{MSE}}_{\mathrm{Sim}2,i}}$$

For the 16 evaluated configurations, a set of values $\delta_{i}$ was obtained. For instance,

$$\delta_{1}\approx 0.0444\;\mathrm{and}\;\delta_{11}\approx 0.02.$$

These results indicate that, for these configurations, the differences between the two simulations are less than 5%. That is, the variation in the MSE metric between Sim. 1 and Sim. 2 is very small, suggesting significant stability in the performance of A under changes in initial conditions.

If A had strongly overfitted to a specific configuration or seed, values of $delta_{i}$ much closer to 1 would have been observed, reflecting a strong dependency on the original run. Instead, the low values obtained confirm that A demonstrates stable and consistent behavior, reducing the likelihood of overfitting under the evaluated conditions. Thus, the numerical evidence supports the conclusion that overfitting is minimal, at least within the stable subset of analyzed configurations. Thus, based on the foregoing evidence, the best overall results for Sim. 1 were achieved with Configuration 12 and for Sim. 2 with Configuration 10. These configurations demonstrate a balance among the learning rate (α), the importance of future rewards (γ), exploration (ϵ with a slow decay rate), and persistence in long-term learning.

As a matter of fact, configurations with α=0.01 exhibited a higher tendency to converge by truncation. This is due to the fact that, despite the balance between exploration and exploitation to identify successful states, the learning rate is very low, which limits the capacity of A to execute optimal actions.

The MSE values remained low because comparisons were made against the current Q table, ensuring that the action *a* taken did not deviate significantly from the best known by A. In contrast, PE values support the notion that a proper balance between exploration and exploitation is essential.

**Figure 6:**
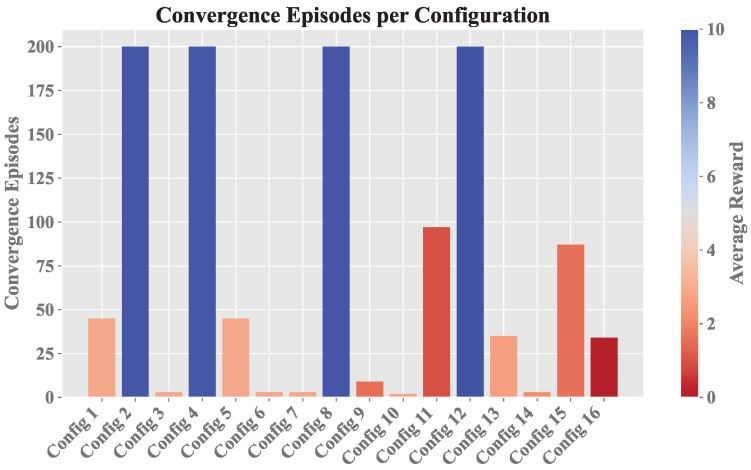
A convergence for hyperparameter configurations.

Based on the similar results of both simulations, the subsequent analyses were carried out based on Sim. 2, since it presented a minor number of episodes terminated by truncation, which would allow for a more detailed study of the agent’s behavior during learning. Figure 6 shows the convergence relationship for each configuration.

Configurations with convergence at 200 indicate that they were truncated and did not achieve stable learning, which is a phenomenon that was more pronounced for the initial α value. It is also observed that the four configurations terminated by truncation had a good reward, and despite this, they are not desired configurations given that there must be a balance between the reward achieved by the agent and also the time it takes to obtain it, mainly in the focus of this work where the efficiency of the proposed architecture is at stake.

Figure 6 also shows the average reward *r* before reaching convergence, highlighting the contrast between the performance of A while exploring new actions $a^{\prime }$ and its performance once it began to prioritize optimal actions. For the second α value, the average reward *r* was initially low before convergence; however, most configurations eventually achieved values close to 10, representing the maximum reward when exploiting any state *s* in $V_{R}$, $V_{I}$, or $V_{E}$.

**Figure 7:**
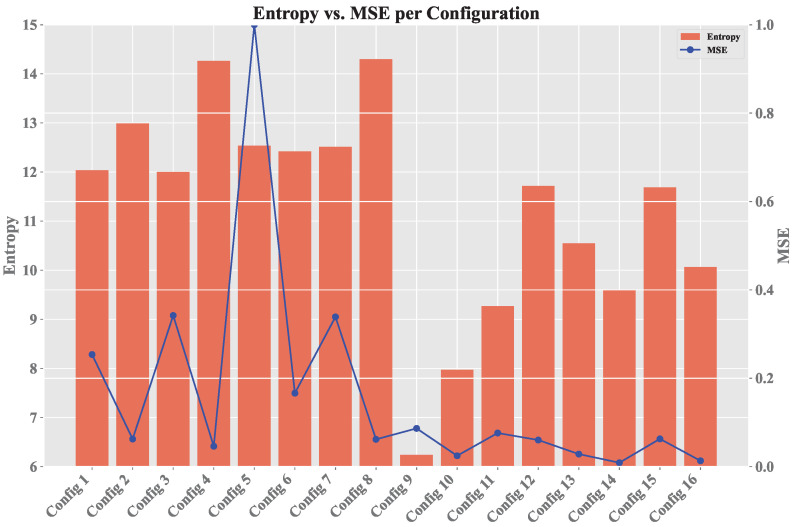
Comparison of PE and MSE for hyperparameter configurations.

Figure 7 displays the relationship between policy entropy $\pi_{\epsilon }$ and MSE for each configuration. Higher PE values indicate greater disorder in the selection of $\left\{a_{1},\cdots ,a_{u}\right\}$, while lower PE values indicate more stable learning, showing that the second learning rate is more consistent. The same applies to MSE, which is generally lower in the final configurations. Peaks in these values suggest the use of exploration with a higher ϵ value, which results in the selection of random actions that may not be optimal, thus affecting the error rate.

To facilitate a detailed comparison, two agent configurations were selected from Sim. 2: Config. 9, which exhibited a balanced performance in terms of reward and steps, and Config. 4, which demonstrated a less favorable outcome. While Figure 5 identifies the optimal configurations for each simulation, these often achieved perfect reward averages, which is an atypical occurrence in such environments. Therefore, we opted for Config. 9 and Config. 4 to provide a more realistic comparison.

**Figure 8:**
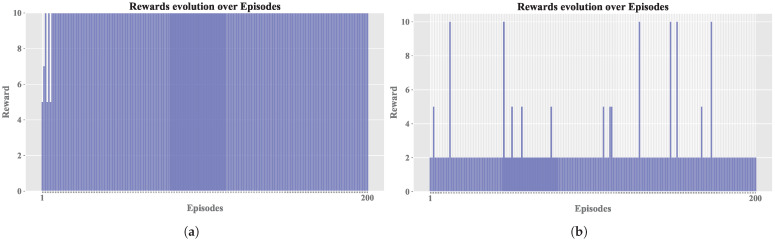
Average reward comparisons. (**a**) Best configuration average reward. (**b**) Worst configuration average reward.

Figure 8 shows the comparison between the cumulative reward of both configurations. It shows how the best configuration achieved the maximum reward in almost all episodes, with the exception of the initial episodes where the agent had not yet identified an optimal operating policy; in contrast, the other configuration had lower rewards throughout the training.

**Figure 9:**
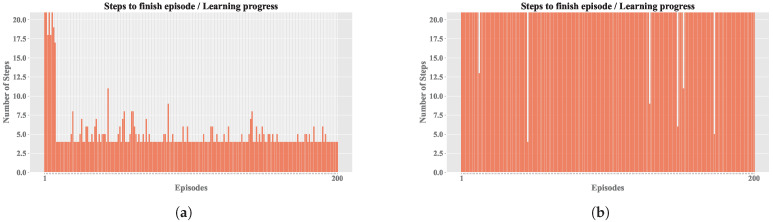
Average EL comparisons. (**a**) Best configuration average EL. (**b**) Worst configuration average EL.

Figure 9 presents comparisons of average episode length (EL) between the configurations. In Figure 9a, the best configuration achieved shorter episodes, indicating faster convergence. In contrast, Figure 9b, the worst configuration, showed longer episodes with no convergence, resulting in episodes being truncated.

**Figure 10:**
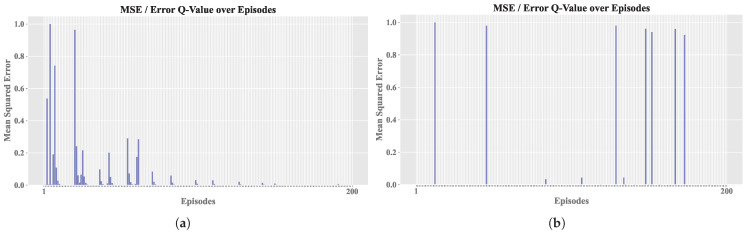
Average mean squared error (MSE) comparisons. (**a**) Best configuration average MSE. (**b**) Worst configuration average MSE.

**Figure 11:**
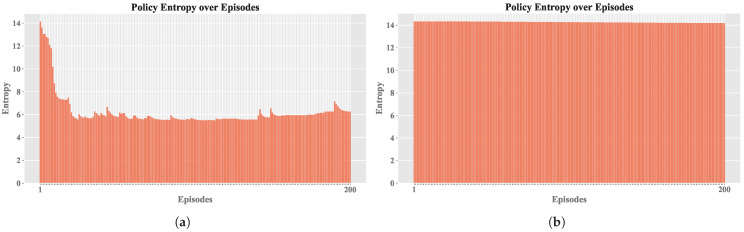
Average PE comparisons. (**a**) Best configuration average PE. (**b**) Worst configuration average PE.

Regarding the EL, as shown in Figure 9, it is evident that training without convergence led to truncation across all episodes (exceeding 20 steps). In contrast, the other configuration achieved early convergence, and occasionally, random actions were selected during training to apply the exploration technique; if these actions prove ineffective, the previously learned actions $a^{\prime }$ are resumed. Figure 10 and Figure 11 share the characteristic that, in the best configuration, an initial phase of training disorder was observed until convergence was reached. Conversely, in the configuration that failed to stabilize, there were higher entropy measures and persistent error spikes throughout the training process.

**Figure 12:**
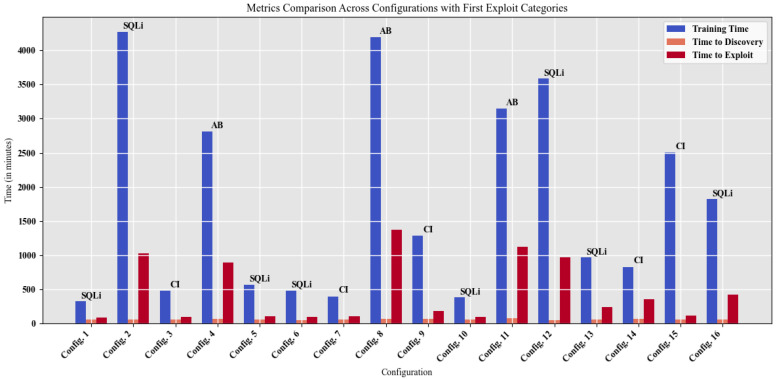
Evolution of the metrics training time, time to discovery, and time to exploit across 16 configurations for two RL simulations. The figure shows how each metric evolves across the configurations, with different colors representing the individual metrics. Additionally, the figure includes the corresponding “First Exploit” categories, highlighting the different attack methods used.

In addition to the metrics associated with RL environments, other metrics typical of a pentesting environment were observed and are described in Figure 12. These metrics provide a broader perspective on the model’s performance by incorporating key aspects relevant to real-world penetration testing scenarios. Specifically, this figure illustrates, for each configuration, the progression of the total training time, the time required to detect vulnerabilities, and the time taken to execute the first successful attack. Furthermore, it identifies the specific type of attack performed, offering insights into the effectiveness and efficiency of different strategies employed during the training process. These additional metrics are essential for evaluating the practical applicability and adaptability of the models in dynamic and complex security environments.

### 4.2. Performance Analysis of BERT QA Models

In the case of BERT QA RL + RS, there are different pre-trained models, with each varying in terms of vocabulary size, weights, attention heads, transformer layers, and embeddings. The selection of models for BERT QA RL + RS is justified by their balance between accuracy and efficiency, with each one being suitable for different scenarios:

- *BERT uncased:* Ref. [44] used this as the base model, providing a reliable benchmark for QA tasks and demonstrating a robust contextual understanding. It consists of 12 transformer blocks *k*, 768 embedding dimensions, 12 attention heads, and 4096 weights *W*.
- *RoBERTa:* Ref. [45] enhanced BERT pretraining by removing certain constraints and leveraging a larger dataset with a more versatile masking approach, offering improved accuracy for QA and making it ideal for maximizing precision. It is composed of 12 transformer layers *k*, 768 embedding dimensions, 12 attention heads, and 3072 weights.
- *DistilBERT:* Ref. [46] employed a lighter version of BERT uncased that retains much of the performance at a lower computational cost, making it ideal for QA environments with limited resources. It includes approximately 60% of the original vocabulary, consisting of 512 embeddings, 12 attention heads, and 3072 weights *W*.

In the context of performance metrics for BERT QA RL + RS, an informative resource analysis can be performed to highlight the computational cost, the cumulative learning progress of BERT models [44,45,46], and the temporal efficiency of the training process, as shown in Table 3.

Since the training was conducted in an intentionally vulnerable test environment, these times are lower than what would be obtained in a real-world environment, but they serve as a basis for evaluating the agent’s performance in the executed environment. From the figure it is possible to obtain the three categories of attacks that the agent manages to perform at the beginning of its training correspond to authentication bypass (AB), SQL injection (SQLi), and command injection (CI).

In this line of reasoning, Table 4 presents a summary of the computational efficiency metrics for each BERT QA RL + RS model, indicating that DistilBERT [46] achieved the fastest training time while maintaining a low loss, which translated into an efficient learning rate.

From the results obtained, the total training time for the system integrating BERT QA RL + RS and RL can be calculated. In this case, the average training time for the RL agent, across all configurations, is 34 min; while the average training time for the BERT models is 1095.4 min, as detailed in the Table 4 (expressed in seconds). Thus, the total training time for the proposed architecture is 1129.4 min.

As for the execution, the total time will depend on the models selected to finally integrate the architecture. However, in general, a time of approximately 2 min is estimated for the inference of the BERT models, to which must be added the execution time of the RL agent with its best configuration (Config. 9–21 min). This last step will be necessary only if the BERT model does not provide an answer.

In terms of resource consumption, both models require at least 16 GB of RAM and 4 CPU cores, which are also necessary for the use of the models during the inference phase.

Thus, the choice of the best model depends on the specific project priorities. If speed and efficiency are critical, DistilBERT is the most suitable choice due to its superior runtime performance and comparable metrics. However, if the project prioritizes slightly higher QA performance, BERT or RoBERTa may be preferred, although the trade-off in computational efficiency should be considered.

### 4.3. Combined Performance Metrics for RL and BERT QA RL + RS

The third component of the RL and BERT QA RL + RS analysis focuses on metrics that evaluate the quality of predictions and inferences made by the models when predicting tuples $\left\{\hat{A_{S}},\hat{A_{E}}\right\}\in \hat{A}$, where *A* represents the ground truth. These metrics are critical for understanding how well the models capture and replicate the expected outputs in tasks requiring precise and contextually accurate answers. Table 5 details these metrics, which provide a comprehensive evaluation of the performance of the models. Additionally, Table 6 summarizes the hyperparameters used during the training of all models to ensure optimal configurations for achieving high-quality results.

The QA accuracy metrics for each model are shown in Table 7.

For this evaluation, weighted average metrics were used, providing a balanced assessment of model performance across all instances in the BERT QA RL + RS task. This approach accounts for the varying importance and distribution of questions and answers, offering a more accurate representation of overall model performance. Since all models demonstrated high performance with metrics exceeding 97% for both the EM and F1-Score, these weighted metrics ensure fair evaluation and reflect performance across different question types.

**Figure 13:**
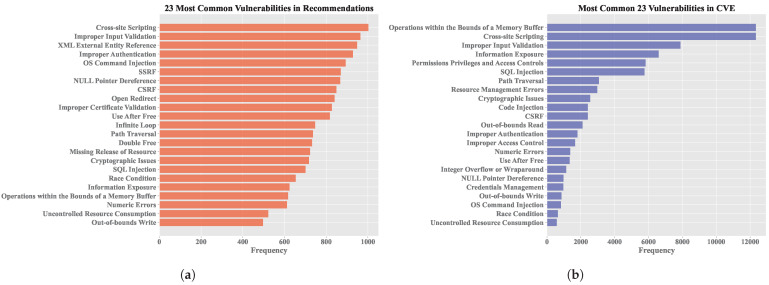
Common vulnerabilities supported by the solution. (**a**) Vulnerabilities in the recommendation system. (**b**) Top vulnerabilities in the CVE dataset.

Since attacks are recommended based on the vulnerabilities of each target machine, it is relevant to analyze the vulnerabilities considered by the recommendation system. Figure 13a shows the list of vulnerabilities available in the recommendation system, while Figure 13b presents the top 23 vulnerabilities in the CVE dataset.

Among the 23 vulnerabilities currently used by the recommendation system, 14 are among the top vulnerabilities according to CVE, indicating that the system is targeting critical and important vulnerabilities and suggesting relevant attacks accordingly.

## 5. Discussions

To delve deeper into the BERT QA RL + RS proposal, this section adopts three comparative approaches to evaluate its performance and contributions. Each approach emphasizes a specific aspect of the architecture, from its qualitative advantages to its computational and statistical underpinnings, as well as its comparison with alternative methodologies. These approaches are structured into three main subsections:

- *Qualitative* Comparison with State-of-the-Art and Common Pentesting Tools (Section 5.1)—This subsection explores the qualitative strengths of the proposed architecture in comparison with existing solutions, focusing on its adaptability, scalability, and modular design.
- *Statistical Validation Analysis and Computational Complexity Comparison* (Section 5.2)—This subsection details a statistical analysis of the proposal’s reliability and contrasts its computational complexity with conventional methods such as Q-Learning and DQN.
- *Comparison of BERT QA RL + RS with Genetic Algorithms* (Section 5.3)—This subsection provides a comparative analysis between the proposed architecture and Genetic Algorithms (GAs), emphasizing their respective strengths and limitations in a penetration testing context.

### 5.1. Qualitative Comparison with State-of-the-Art and Common Pentesting Tools

In this first point of comparison, the proposed BERT QA RL + RS framework is evaluated alongside traditional penetration testing tools and AI-enhanced solutions. These tools include widely recognized frameworks such as Metasploit [47], Nessus [48], OWASP ZAP [49], and Burp Suite [50], as well as advanced AI-enhanced tools like PentestGPT [51] and CyberProbe AI [52]. The comparison is organized based on the five phases of penetration testing outlined in NIST 800-155: preparation, discovery, analysis, exploitation, and reporting.

The strengths and limitations of each tool are presented, emphasizing their specific contributions and gaps in comprehensive penetration testing compared with BERT QA RL + RS, as listed in Table 8.

The analysis summarized in Table 8 indicates that BERT QA RL + RS demonstrates robust capabilities in addressing complex, multi-layered systems through automated, end-to-end testing processes. Its reinforcement learning and contextual question-answering methodology enable dynamic adaptation to evolving environments and effective prioritization of vulnerabilities based on criticality. In comparison, traditional tools such as Metasploit and Nessus, while reliable in specific areas, require significant manual configuration for advanced tasks and lack comprehensive automation. Similarly, AI-enhanced solutions like PentestGPT and CyberProbe AI provide notable automation capabilities but encounter limitations when addressing complex or ambiguous scenarios, which are areas where the BERT QA RL + RS framework shows a distinct advantage.

The second point of comparison evaluates the effectiveness of the proposed architecture, BERT QA RL + RS, in identifying and recommending effective attacks. The BERT QA RL +RS framework demonstrated an accuracy rate exceeding 97%, highlighting its robustness in handling scenarios that, despite being based on two virtual machines, incorporate diverse and realistic configurations emulating highly complex cybersecurity environments.

The VMs employed in this study are designed to simulate diverse and realistic configurations, encompassing exploitation paths such as network services and web vulnerabilities. These environments also incorporate dynamic configurations, including interconnected services, weaknesses in ABs and cryptography, and scenarios representative of real-world production systems. For example, the setups include services specifically configured to allow for PEsc attacks and ID, which are common challenges encountered in operational environments.

The first VM is based on Linux 20.04 and incorporates the Metasploitable 2 framework, offering over 100 exploitation paths, 50 CVEs with proof-of-concept exploits, and more than 40 documented weaknesses from CWE. This setup includes services vulnerable to remote CI, misconfigurations, BFA, and CF. The second VM extends this environment, including Windows 8 and 10 systems alongside services such as Tomcat, Jenkins, and Python’s Flask, providing 80 paths for reconnaissance, vulnerability analysis, and exploitation and integrating 40 CVEs with over 50 CWEs.

Through these configurations, the BERT QA RL + RS architecture demonstrates its capacity to manage scenarios with diverse and realistic vulnerability profiles. Its modular design and contextual processing capabilities enable it to extend beyond the current test environment of two VMs, supporting extrapolation to more complex setups, such as cloud networks, containerized systems, and IoT devices. This scalability makes the architecture adaptable to evolving cybersecurity landscapes while maintaining a systematic approach to vulnerability assessment.

Table 9 presents a detailed comparative overview of the capabilities of BERT QA RL + RS in relation to state-of-the-art approaches. This comparison includes the methods used for data ingestion, the types of environments supported, and the scalability of each approach in addressing complex scenarios. Additionally, the table highlights specific exploitation capabilities such as network services, web vulnerabilities, and misconfiguration analysis, alongside adaptability to dynamic environments. This comprehensive breakdown underscores the modularity, contextual adaptability, and versatility of the proposed architecture in comparison to existing methods.

In terms of computational complexity, BERT QA RL + RS notably improves the agent’s ability to navigate intricate scenarios. Unlike previous models limited by rigid action–reward frameworks, as seen in studies [15,16], this approach leverages a BERT-based system to interpret detailed vulnerability descriptions and dynamically suggest a broader array of attacks.

Although the environment comprises only two VMs, it incorporates over 100 exploitation paths, 90 CVEs, and 50 CWEs simulating interconnected services, AB weaknesses, CFs, and production-like configurations. These include scenarios such as web applications, network services, PEsc and CF, demonstrating that the complexity of the scenarios arises from their internal richness rather than the number of nodes.

Compact environments such as the two VMs provide significant advantages in reinforcement learning. As highlighted in [53], environments with high-dimensional continuous state spaces often result in inefficiencies due to redundant observations, which can lead to costly policies and actions. The incremental complexity of the two VMs enables the RL estimator to progressively adapt to diverse configurations, avoiding unnecessary overhead and allowing the development of stable, scalable policies. This design aligns with established practices such as NASim [20] and PenGym [21], which also employ basic environments to train RL agents effectively.

In contrast to studies like [15,16], which required 5 h for networks of size 10 and 100 h for networks of size 50, BERT QA RL + RS achieved an average completion time of 0.55 h. This sharpens the distinction with approaches such as those in [9,10,19,22], where Deep RL-based agents were found to necessitate high-computation environments to execute attack sequences. By processing contextual information dynamically, BERT QA RL + RS reduces unnecessary exploration and improves computational efficiency, enabling successful adaptation to more complex scenarios such as cloud networks, container systems, and IoT environments.

Moreover, BERT QA RL + RS directly addresses critical challenges in penetration testing, such as the need for rapid and efficient responses to vulnerabilities within diverse infrastructures. While traditional methods often struggle with scalability and fail to adapt to rapidly changing cybersecurity landscapes, this approach makes notable advances. For instance, study [9] introduced DDQNs to enhance observational capabilities, but integrating DistilBERT allows for a deeper contextual understanding, leading to faster and more accurate results in scenarios where nuanced interpretation of security data is essential.

### 5.2. Statistical Validation Analysis and Computational Complexity Comparison

A statistical analysis was conducted to validate the effectiveness and reliability of the proposed BERT QA RL + RS framework. Unlike most state-of-the-art approaches in reinforcement learning applied to pentesting (e.g., Yi et al. [9], Hamidi et al. [10], Ghanem [15], Ghanem [16], Zennaro et al. [17], Chaudhary et al. [18], Nhu et al. [19], Schwartz and Kurniawati [20], Tran et al. [22], Nguyen et al. [21], and Wei et al. [23]), previous research often reports substantial improvements—such as enhanced efficiency, scalability, or adaptability—without employing nonparametric statistical tests to confirm that observed differences are not due to chance.

In this study, the Wilcoxon Signed-Rank (WSR) test [54] was used to evaluate the statistical significance of performance differences between the complete proposal (BERT QA RL + RS with Q-Learning) and the baseline configurations (Q-Learning only and DRL only). The WSR test, which does not assume normality, is appropriate for complex, nonparametric data. Achieving *p*-values < 0.05 in all comparisons indicates that the improvements are statistically significant and not merely random fluctuations. The WSR test follows these steps:

1. *Calculating Differences ($D_{i}$)*: For each paired observation, determine the difference in execution times of the two compared configurations, $D_{i}=T_{1,i}-T_{2,i}$.
2. *Sorting Absolute Differences*: Arrange $|D_{i}|$ in ascending order and assign ranks.
3. *Summation of Ranks*: Separate ranks into those associated with positive ($D_{i}>0$) and negative ($D_{i}<0$) differences:
  $$W^{+}=\sum_{D_{i}>0}R_{i},\;W^{-}=\sum_{D_{i}<0}R_{i}$$
4. *Test Statistic*: The test statistic W is the smaller of $W^{+}$ and $W^{-}$.
5. *Determining the p-Value*: Compare W against the Wilcoxon distribution at α=0.05. If p<α, reject the null hypothesis.

By applying the WSR test, the analysis confirms that the proposed architecture’s enhancements are not attributable to random variation. While previous studies highlight a variety of improvements, the absence of rigorous statistical validation in those works leaves uncertainty as to whether their observed gains are statistically significant. In contrast, the WSR-based results here not only verify the superiority of BERT QA RL + RS but also provide statistically robust evidence.

The evaluation encompassed the following comparisons:

- *Complete Proposal (BERT QA RL + RS with Q-Learning) vs. Q-Learning only*: Demonstrates significant improvement, ensuring that integrating BERT QA RL + RS yields measurable, nonrandom gains.
- *Complete Proposal vs. DRL only*: Statistically confirms that the proposed approach outperforms DRL in terms of efficiency and adaptability.
- *Q-Learning only vs. DRL only*: Shows that even a standard Q-Learning strategy statistically surpasses DRL, offering a baseline from which the proposed method’s additional gains can be contextualized.

Table 10 presents the WSR results, revealing statistically significant differences favoring the complete proposal.

The importance of the WSR-based validation is further illustrated by contrasting these statistically confirmed results with the state-of-the-art approaches listed in Table 11, which presents a comparative evaluation of the eleven referenced studies. While these studies report various forms of improvement—such as faster convergence, increased scalability, or better handling of complex vulnerabilities—only three (Zennaro et al., Tran et al., and the proposed framework) have incorporated statistical tests to validate their findings. Most studies do not include a nonparametric statistical assessment like the WSR test, leaving open the possibility that their reported improvements may be attributable to randomness.

In other words, although prior works demonstrate promising advancements, the lack of rigorous statistical validation raises questions about the reliability of their reported gains. By introducing WSR-based statistical validation, the proposed framework elevates its demonstrated improvements beyond anecdotal or heuristic evidence. This methodological advancement ensures that the observed enhancements are not only qualitative or isolated, but are instead supported by objective, statistically sound measures.

In summary, the relationship between the WSR results and the comparative evaluation against state-of-the-art approaches lies in the establishment of a new methodological standard. While the referenced studies indicate potential advantages, none employed a nonparametric test like WSR to substantiate their claims statistically. By contrast, the results presented here not only highlight superior performance but do so with the statistical rigor necessary to confirm that these improvements are both real and significant.

Additionally, since the presented proposal applies Q-Learning in scenarios requiring agent execution, a complexity evaluation of the BERT QA RL + RS system was conducted in comparison to traditional DRL methods, such as the DQN algorithm, which is commonly used for addressing penetration testing tasks. This comparison illustrates how the proposed system, as the environment increases in complexity and dimensionality, optimizes its performance, demonstrating its ability to manage larger and more intricate environments with greater efficiency.

*Q-Learning*: The complexity of the Q-Learning algorithm can be expressed in terms of the number of states *S* and the number of actions *A* available. The Q-table has a size of S×A, and updating it requires traversing all combinations of states and actions, resulting in the complexity shown in Equation (20).

(20) Q-Learning=O(S×A)

*DQN*: The complexity of the DQN depends on the number of states $\left\{s_{1},\cdots ,s_{u}\right\}$, the number of actions $\left\{a_{1},\cdots ,a_{u}\right\}$, the number of layers *L*, and the number of neurons per layer *N*. In a DQN, the neural network processes each state and generates a distribution over the actions. Assuming forward propagation and backpropagation have a complexity of $O(L\times N^{2})$, and this process is repeated for each state and action, it can express the complexity in terms of states and actions, as shown in Equation (21).

(21) $$\mathrm{DQN}=O(S\times A\times L\times N^{2})$$

In addition to the Q-Learning complexity, the training complexity of the BERT-based RS is included, as shown in Equation (22). Here, *D* represents the number of elements in the training dataset, and *T* represents the number of allowed tokens. Training complexity has been used, since the RS has linear complexity for inference.

(22) BERTQARL+RS+RL=O(S×A+D×T)

This demonstrates that BERT QA RL + RS maintains a significantly lower complexity compared to DQN. This factor becomes crucial in penetration testing applications, where a high number of layers and neurons can hinder the practical implementation of DQN. Moreover, it is important to note that the Q-Learning complexity applies only once during the process, whereas other approaches may incur higher computational costs for each evaluated machine, making BERT QA RL + RS more scalable and efficient in practice.

Performance comparisons further reveal that similar RL agent designs, such as in study [17], reported convergence in 100 and 2000 episodes for web hacking scenarios. By comparison, the best configuration in BERT QA RL + RS converged within just 12 episodes, significantly improving efficiency. This trend continued with studies [10,20], where Q-Learning models required approximately 1000 episodes to converge and DL-based models around 100, indicating that while DL can offer faster convergence, it demands more computational resources. The BERT-based QA system in BERT QA RL + RS reduces computational demands while maintaining strong accuracy, setting a balance between effectiveness and efficiency.

In [18], traditional Q-Learning was applied to post-exploitation phases with a high computational cost, as the model focused on compromised environments, where it converged on plaintext credential discovery and PEsc tasks. Although effective, this approach has limitations when applied to environments with broader exploitation needs. In contrast, BERT QA RL + RS leverages the RS to assess vulnerabilities dynamically, thus supporting a broader range of exploits without the need for specific pre-compromised environments.

From a Deep Learning perspective, studies in [10,19] suggest that classifiers for exploitation environments require considerable computational resources. In [19], output layer generalization guided the agent’s RL process, while [10] used standard tools to detect optimal conditions. These models’ reliance on high-resource DL architectures makes them more costly than BERT QA RL + RS, which achieves similar effectiveness by integrating simpler Q-Learning with BERT’s language capabilities, making it efficient for practical use.

Further, within complex penetration testing environments, projects such as Network Attack Simulator (NASim) [20] and PenGym [21] simulate multiple phases of penetration testing with intricate environments. The RL model used by NASim operates based on high-cost kill chains, while PenGym enhances efficiency through session-based modules. However, both frameworks are costly in practice due to their complex dependencies on network configurations. The BERT QA RL + RS system, with its streamlined architecture, achieves a balance between accuracy and computational cost, making it more adaptable to real-world applications.

The complexity of CRLA in [22] is well-suited for discrete action spaces, though its approach to cascading agents increases complexity exponentially as scenarios scale, limiting applicability in resource-constrained settings. In comparison, BERT QA RL + RS offers a more computationally sustainable solution by using a single, cohesive agent equipped with BERT’s language modeling capabilities, allowing for efficient action selection without exponential growth in complexity.

**Figure 14:**
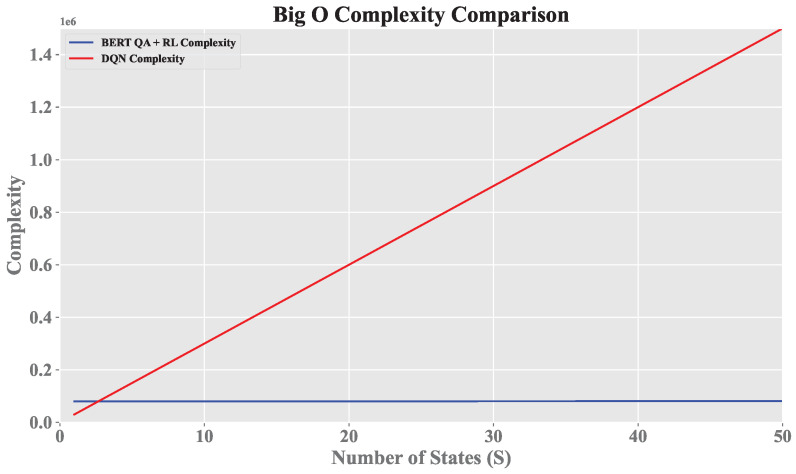
Big O Complexity Comparison between the proposed method and DQN algorithms.

Figure 14 shows the growth of both expressions as a function of the increase in the number of states in the environment, with constants A=15, L=5, N=20, D=400, and T=200.

This comparison has been made with a small neural network example, although in practice for penetration testing tasks, these networks are typically much larger, which would increase the multiplicative factor and, consequently, the training complexity. Additionally, a dataset with few elements and few tokens for the RS has been considered. In Figure 14, it is observed that for the initial values of *S*, the DQN model offered lower complexity; however, as the number of states grew, BERT QA RL + RS remained lower, while DQN complexity increased rapidly.

Regarding comparisons made with Q-Learning, the DQN and existing work from the literature have highlighted the potential of the proposal, showing how, as the complexity and dimensionality of the environment increases, the performance of the solution improves. However, when considering these results, it is critical to recognize some inherent limitations of the approach used. The RL agent’s reward structure was designed to streamline the learning process by focusing on a structured sequence of states, such as reconnaissance, vulnerability analysis, and exploitation. This approach ensures measurable progress and simplifies training in controlled scenarios. However, this design choice does not fully capture the complexity of real-world pentesting, where paths are often dynamic and contingent upon factors such as the discovery of unexpected vulnerabilities, system configurations, or opportunities for lateral movement. Future iterations of the model could benefit from incorporating reward mechanisms that adapt to these dynamic and nonlinear paths, enabling a more realistic representation of adversarial behavior.

Another limitation is the use of intentionally vulnerable environments such as Metasploit, which can reduce the agent’s ability to scan for security breaches. In future research, it is planned to extend this approach to more diverse and dynamic environments to address the unpredictability and complexity inherent in real-world networks, thereby improving the robustness of the system against false positives and other challenges commonly observed in real-world penetration testing situations.

### 5.3. Comparison of BERT QA RL + RS with Genetic Algorithms

A key comparative aspect emerges from the examination of relationships between RL and Genetic Algorithms (GAs), given that both paradigms iteratively search solution spaces in a manner analogous to the human aptitude for experiential generalization [55]. In consonance with [56], GAs rely on fitness models to evaluate populations of candidate solutions, employing operators such as selection, crossover, and mutation without requiring a sequential notion of states or actions. Mathematically, considering a population $P=\{x_{1},x_{2},\cdots ,x_{n}\}$ and a fitness function f(x), GAs iteratively generate new populations $P^{\prime }$ by combining and mutating selected individuals based on f(x), thus striving to maximize or minimize f(x) without temporal dependencies or cumulative rewards.

However, in contrast to RL, GAs differ notably in their feedback mechanism. RL continuously receives sequential rewards by interacting with a dynamic environment, seeking a policy π that maximizes the expected value of accumulated rewards $E\left[\sum_{t}\gamma^{t}r_{t}\right]$, where γ is a discount factor and $r_{t}$ represents the reward at time *t*. Conversely, GAs optimize a fitness function over a population of solutions, refining them without the necessity of incremental rewards or environmental interaction, thus improving the global quality of solutions through pointwise evaluations.

In Section 4, a canonical penetration testing scenario was described that aligned with NIST SP 800-115 guidelines. Here, the pentester initiates the process with limited information about the target, conducting reconnaissance, vulnerability identification, and exploitation, and finally documenting findings and recommendations. Within this context, the BERT QA RL + RS architecture proves pivotal: RL adapts its actions as it receives rewards from a dynamic environment, while BERT contributes semantic and contextual understanding. This sharply contrasts with what GAs might achieve; although they could, in theory, generate multiple solutions (e.g., by combining various attack vectors), GAs lack the intrinsic sequential feedback mechanism required for dynamic policy updates based on the environment’s state.

Below in Table 12, a comparative table is presented, drawing upon the scenario described to illustrate the advantages and disadvantages of pure RL, GAs, and the integrated BERT QA RL + RS approach. The table highlights that, while a GA could be theoretically applied to penetration testing challenges, its capacity to adapt to environmental changes and leverage contextual knowledge (such as CVE, CWE, and textual recommendations) is considerably more limited than that of RL or the integrated BERT QA RL + RS solution.

## 6. Conclusions

The proposed BERT QA RL + RS architecture, which integrates reinforcement learning with a recommendation system leveraging contextual data, presents a significant advancement in cybersecurity assessment. This system addresses the critical need to identify vulnerabilities and strengthen preventive controls by simulating real-world malicious behaviors within Information Technology (IT) frameworks. Achieving an accuracy and efficiency exceeding 97%, BERT QA RL + RS delivers precise and relevant penetration testing processes without requiring agent retraining, thereby reducing the time and resources typically associated with such complex environments. Its adaptability across diverse contexts further enhances its potential for proactive vulnerability detection and effective threat mitigation.

The inclusion of DistilBERT in the recommendation system elevated the contextual relevance of suggested attack strategies, underscoring the transformative role of large language models in cybersecurity. By providing rich, context-aware recommendations, this approach not only enhances the precision of security assessments but also establishes a foundation for integrating advanced ML techniques to reduce false positives—an enduring challenge in penetration testing.

Future research should investigate deep learning methodologies to further improve system detection accuracy and enhance adaptability to unpredictable cybersecurity environments. Evaluating the system within practical, real-world workflows is an essential next step, particularly by incorporating feedback from human pentesters as part of a continuous learning process. This could significantly refine system recommendations, aligning them more closely with evolving operational demands.

Furthermore, examining how human expertise contributes to decision making, prioritization of vulnerabilities, and system adaptability, represents a promising area of study. Insights gained from how human testers interpret and utilize system outputs in operational settings would inform refinements, ensuring greater usability and alignment with the complexity of penetration testing practices.

Embedding a feedback loop where human inputs validate, calibrate, or challenge system outputs would enable higher precision and contextual relevance. Such an approach bridges the gap between automated solutions and expert-driven assessments, ensuring the system remains effective and relevant amidst the increasingly complex cybersecurity landscape.

Future work should also address the inherent challenges of applying reinforcement learning to incremental and unpredictable environments, where state–action spaces ({s,a}) expand dynamically over time. One potential approach to tackle this limitation is the development of hierarchical reinforcement learning (HRL) frameworks, which decompose complex tasks into subtasks with localized state–action spaces. By integrating HRL with transfer learning techniques, the system could adapt previously learned policies to evolving scenarios, effectively managing the growing complexity of production environments. Such an advancement would further enhance the system’s applicability to real-world cybersecurity challenges.

In addition, developing self-learning and continuous adaptation mechanisms is essential for maintaining system resilience against emerging vulnerabilities and attack methods. Comparative analyses with other security assessment methodologies across diverse domains, including critical infrastructure and enterprise security, would validate the system’s effectiveness and competitive advantages. These evaluations will position BERT QA RL + RS as a versatile and impactful tool, meeting the modern challenges of cybersecurity with precision and adaptability.

## Figures and Tables

**Table 1:** Description of hyperparameters for A and their respective values.

Hyperparameter	Description	Value 1	Value 2
Alpha (α)	Learning rate that controls how much the agent learns from each new experience. A higher value accelerates learning but may lead to unstable convergence.	0.01	0.5
Gamma (γ)	Discount factor that determines the importance of future rewards. A higher value prioritizes long-term rewards.	0.9	0.5
Epsilon (ϵ)	Exploration rate that controls the probability of the agent taking a random action instead of following its policy. A higher value encourages exploration.	0.2	0.015
Epsilon Decay ($\epsilon_{decay}$)	Decay rate for the exploration rate (ϵ), which controls how ϵ decreases over time, allowing the agent to reduce exploration as it learns.	0.999	0.9

**Table 2:** A configuration parameters.

Configuration	α	γ	ϵ	$\epsilon_{\mathrm{decay}}$
Config. 1	0.01	0.9	0.2	0.999
Config. 2	0.01	0.9	0.2	0.9
Config. 3	0.01	0.9	0.015	0.999
Config. 4	0.01	0.9	0.015	0.9
Config. 5	0.01	0.5	0.2	0.999
Config. 6	0.01	0.5	0.2	0.9
Config. 7	0.01	0.5	0.015	0.999
Config. 8	0.01	0.5	0.015	0.9
Config. 9	0.5	0.9	0.2	0.999
Config. 10	0.5	0.9	0.2	0.9
Config. 11	0.5	0.9	0.015	0.999
Config. 12	0.5	0.9	0.015	0.9
Config. 13	0.5	0.5	0.2	0.999
Config. 14	0.5	0.5	0.2	0.9
Config. 15	0.5	0.5	0.015	0.999
Config. 16	0.5	0.5	0.015	0.9

**Table 3:** Computational efficiency metrics.

Metric	Description	Formula
Total FLOPs	Represents the computational workload during model training, providing an indication of resource consumption.	—
Training Loss	Reflects the model’s progress in learning by measuring the discrepancy between predicted and actual values.	$L=\frac{1}{N}\sum {\left(A-\hat{A}\right)}^{2}$
Training Time	Represents the total duration of the training process, indicating the temporal efficiency of the model training.	—
Training Samples per Second (SPS)	Indicates the rate at which data samples are processed, measured in samples per second.	$SPS=\frac{\mathrm{Total}\;\mathrm{Samples}}{\mathrm{Training}\;\mathrm{Time}}$
Training Steps per Second (STS)	Denotes the frequency of training steps, providing insight into the model’s training step speed.	$STS=\frac{\mathrm{Total}\;\mathrm{Steps}}{\mathrm{Training}\;\mathrm{Time}}$

**Table 4:** Computational efficiency metrics for BERT QA training.

Model	FLOPs	Loss	Runtime (s)	SPS	STS
BERT uncased [44]	$3.54\times 10^{10}$	0.000100	77,846.59	0.18	0.012
RoBERTa [45]	$3.53\times 10^{10}$	** 0.000000 **	77,985.63	0.19	0.011
DistilBERT [46]	** $1.76\times 10^{10}$ **	0.004300	**41,347.49**	** 0.35 **	** 0.022 **

**Table 5:** QA accuracy metrics for BERT QA RL + RS.

Metric	Description	Formula
Precision	Measures the proportion of correct words in the prediction relative to all predicted words. In the QA context, it evaluates the accuracy of the model’s generated answer.	$\mathrm{Precision}=\frac{\mathrm{Number}\;\mathrm{of}\;\mathrm{correct}\;\mathrm{words}\;\mathrm{in}\;\hat{A}}{\mathrm{Total}\;\mathrm{words}\;\mathrm{in}\;\hat{A}}\times 100$
Recall	Measures the proportion of correct predicted words relative to all words in the correct answer. Evaluates if the model captures the keywords of the expected response.	$\mathrm{Recall}=\frac{\mathrm{Number}\;\mathrm{of}\;\mathrm{correct}\;\mathrm{words}\;\mathrm{in}\;\hat{A}}{\mathrm{Total}\;\mathrm{words}\;\mathrm{in}\;A}\times 100$
Exact Match (EM)	This metric measures the percentage of answers that exactly match the correct answer. It is a very strict metric, counting answers as correct only if they are identical to the expected response.	$\mathrm{EM}=\frac{\mathrm{Number}\;\mathrm{of}\;\mathrm{correct}\;\hat{A}\;\mathrm{answers}}{\mathrm{Total}\;A\;\mathrm{questions}}\times 100$
F1-Score	F1 is a metric that combines precision and recall. It is used to measure the overlap between predicted words and words in the correct answer. Unlike EM, it does not require exact identity but assesses how many words in the prediction match those in the correct answer.	$F1\text{-}\mathrm{Score}=2\times \frac{\mathrm{Precision}\times \mathrm{Recall}}{\mathrm{Precision}+\mathrm{Recall}}$

**Table 6:** Training hyperparameters for BERT QA RL + RS models.

Hyperparameter	Description	Value
train_epochs	Number of complete passes through the entire training dataset. A higher number of epochs may improve model performance, though an excessive number could lead to overfitting.	3
train_batch_size	Determines the number of samples the model processes simultaneously during training. A larger batch size can accelerate training but requires more memory.	16
eval_batch_size	Similar to the training batch size, it controls the number of samples the model processes at once during evaluation.	16
learning_rate	Defines the rate at which the model adjusts its weights based on the loss gradient. A high learning rate speeds up training but may hinder convergence, while a lower rate results in more stable, albeit slower, learning.	$2\times 10^{-5}$
weight_decay	A regularization parameter that helps prevent overfitting by penalizing large weights, ensuring that the model generalizes well to unseen data.	0.01

**Table 7:** Accuracy metrics comparison for BERT QA models.

Model	Exact Match (EM) (%)	Precision (%)	Recall (%)	F1-Score (%)
BERT [44]	97.5%	98.0904%	98.4848%	98.0146%
RoBERTa [45]	** 99.9998% **	** 99.9999% **	** 99.9998% **	** 99.9999% **
DistilBERT	99.8763%	99.9057%	99.8763%	99.8772%
**Average** [46]	99.1253%	99.3320%	99.4536%	99.2972%

**Table 8:** Comparison of penetration testing tools based on NIST 800-155 methodology.

Tool	Advantages	Disadvantages	NIST 800-155 Coverage
Metasploit [47]	Comprehensive exploitation capabilities; extensive module library for payloads and post-exploitation.	Lacks automation; requires skilled operators; limited discovery and reporting.	Partial: Focused on exploitation and reporting.
Nessus [48]	Robust vulnerability scanning; extensive plugin support.	Limited exploitation features; requires external integration for advanced reporting.	Partial: Emphasizes discovery and analysis.
OWASP ZAP [49]	Highly effective for web application scanning; CI/CD integration.	Limited for multi-layered systems; manual intervention needed for reporting.	Partial: Focused on discovery and analysis.
Burp Suite [50]	Customizable for web penetration testing; rich plugin ecosystem.	Requires significant manual effort; limited to web applications.	Partial: Focused on discovery and analysis.
PentestGPT [51]	AI-based approach; rapid vulnerability identification; generates remediation suggestions.	Limited in complex system architectures; struggles with adaptive learning.	Partial: Covers preparation and discovery.
CyberProbe AI [52]	Advanced AI-driven scanning; effective for threat prioritization; integrates seamlessly with DevSecOps pipelines.	Expensive licensing; relies on pre-trained models; limited exploit generation.	Partial: Focuses on preparation, discovery, and reporting.
BERT QA RL + RS (This proposal)	Fully automated end-to-end framework; reinforcement learning ensures adaptability; QA provides contextual understanding; excels in multi-layered system testing.	Higher resource demands; training requires significant time.	Complete: Covers all NIST phases, including preparation, discovery, analysis, and exploitation.

**Table 9:** Comparison of BERT QA RL + RS with state-of-the-art approaches in terms of data ingestion methods, supported environments, feature coverage, and scalability to complex scenarios.

Work	Data Ingestion Method	Environment Supported	Network Services	Web Vulnerabilities	Misconfiguration Scenarios	Scalability to Complex Scenarios
Yi, J. and Liu, X. [9]	Leverages MulVAL attack graphs and predefined vulnerabilities.	Simulated networks with hosts and subnets.	🗸			Capable of scaling to subnet-based configurations but limited by fixed graph structures.
Hamidi, M., et al. [10]	Connects with tools like Metasploit, SQLmap, and Weevely via APIs.	Controlled setups with predefined exploitation paths.	🗸	🗸		Limited adaptability due to predefined tools and static environments.
Ghanem, M. and Chen, T. [15]	Analyzes penetration testing expert behavior using logs from servers, databases, and routing devices.	Simulated environments with predefined vulnerability paths.	🗸			Limited due to static and predefined scenarios.
Ghanem, M. and Chen, T. [16]	Processes state and action spaces with probabilistic representations of devices and networks.	Networks with devices modeled probabilistically for vulnerabilities.	🗸		🗸	Constrained by reliance on probabilistic state-space representations.
Zennaro, F., et al. [17]	Uses Q-learning to train agents in Capture the Flag scenarios.	Simplified scenarios with predefined port vulnerabilities.	🗸			Restricted to predefined attack paths and ports.
Chaudhary, S. et al. [18]	Employs DT scripts and Python-based log analysis for vulnerability identification.	Focused on file exploitation in predefined Windows and Linux environments.			🗸	Restricted to static environments, without provisions for scalability or dynamic updates.
Nhu, N., et al. [19]	Employs Docker-based environments for training reinforcement learning agents.	Dockerized setups with a variety of CVEs.	🗸	🗸		Scales moderately well but lacks contextual processing for extrapolation.
Schwartz, J. and Kurniawati, H. [20]	Focuses on Metasploit-based testing for FTP vulnerabilities.	Single-port FTP exploitation scenarios.	🗸			Minimal scalability beyond basic vulnerability testing.
Tran, K., et al. [22]	Implements Cascaded Reinforcement Learning Agents for discrete action spaces.	Simulated networks with multiple subnets and hosts.	🗸		🗸	Highly scalable in subnet-based scenarios but less effective in dynamic configurations.
Nguyen, H., et al. [21]	Implements action spaces using Metasploit modules for scanning, exploitation, and PEsc.	Simulations with connected hosts and service vulnerabilities like CVE-2021-41773 and CVE-2015-3306.	🗸			Limited to predefined Metasploit actions and lacks dynamic adaptability to emerging or IoT environments.
Ying, W. et al. [23]	Analyzes and filters CVE data with NLP techniques for event extraction, covering vulnerabilities from 1999 to 2021.	Employs a database of 4638 vulnerabilities from CVE with detailed categorization of 16 CWE types.		🗸		Limited to textual analysis and lacks integration with reinforcement learning or adaptive exploration.
BERT QA RL + RS (This proposal)	Combines BERT’s contextual processing with reinforcement learning for adaptive exploration, integrating real-time data updates for dynamic environments.	Supports diverse configurations, including interconnected services, AB weaknesses, CFs, and real-world scenarios.	🗸	🗸	🗸	Highly scalable due to its modular design, contextual adaptability, and ability to generalize policies across complex environments like cloud and IoT systems.

**Table 10:** Statistical evaluation results of scenario comparisons, with α=0.05.

Comparison	Median Values (Seconds)	Interpretation	W Statistic	*p*-Value
Complete Proposal vs. Q-Learning	97.5 vs. 404.25	Significant improvement	Positive differences dominate	0.027
Complete Proposal vs. DRL	97.5 vs. 604.10	Significant improvement	Positive differences dominate	0.004
Q-Learning vs. DRL	404.25 vs. 604.10	Significant improvement	Positive differences dominate	0.004

**Table 11:** Comparative evaluation with state-of-the-art studies.

Work	Reported Advantage	Main Statistical Disadvantage	Statistical Test Reported
Yi et al. [9]	Scalability	Potential randomness in results	–
Hamidi et al. [10]	Adaptable reward scheme	Unverified significance of improvements	–
Ghanem [15]	PT model understanding	Lack of robustness evaluation	–
Ghanem [16]	Complex RL policies	Unconfirmed performance reliability	–
Zennaro et al. [17]	Handling complex CTF structures	Limited sample size	🗸
Chaudhary et al. [18]	Optimal exploitation routes	No evaluation of statistical significance	–
Nhu et al. [19]	Scalability	Unvalidated reproducibility of results	–
Schwartz and Kurniawati [20]	Extensive simulation scaling	Possible overfitting in simulations	–
Tran et al. [22]	Large action space handling	Limited statistical scope of validation	🗸
Nguyen et al. [21]	Realistic training environments	Unverified scalability of results	–
Wei et al. [23]	Event extraction accuracy	No robust statistical comparisons	–
BERT QA RL + RS (This proposal)	Comprehensive improvements	None	🗸

**Table 12:** Comparison among RL, GAs, and BERT QA RL + RS in a penetration testing scenario aligned with NIST SP 800-115.

Criterion	RL	GAs	BERT QA RL + RS
Nature of Environment	Dynamic, sequential, with rewards tied to actions	Nonsequential, evaluating solution populations without temporal feedback	Dynamic and sequential; integrates RL rewards and BERT’s semantic context
Continuous Adaptation	Adjusts its policy as the environment evolves (new ports, vulnerabilities)	Difficult; changes require new populations and generations without guaranteed rapid adaptation	Iterative adjustment: RL adapts to novel findings, BERT recalibrates responses, incorporating new Q,C,A
Contextual Information	Can leverage structured information (states, rewards) but limited semantic comprehension	No semantic understanding; only evaluates solution fitness without linguistic context	Incorporates BERT’s contextual comprehension, correlating vulnerability descriptions (CVE/CWE) with NIST methodology
Alignment with NIST SP 800-115	RL can implement the cycle (reconnaissance, identification, exploitation) by maximizing rewards at each phase	No natural integration with these phases. GAs optimize a fitness function, lacking a sequential flow suited to recommended stages	Aligns with phases (planning, reconnaissance, vulnerability assessment, exploitation, reporting), leveraging RL and BERT’s semantics
Scalability	Scalable, though potentially requires more computation as complexity increases	Scalable in exploration, but lacks a mechanism guiding adaptive policy changes over time	Scalable; each RL insight is integrated by BERT, facilitating the reuse and expansion of the knowledge base
Final Outcome	An optimal (or near-optimal) policy guiding sequential pentesting actions	A set of candidate solutions without guaranteeing dynamic adaptation or contextual integration	A dynamic policy, informed by semantic context and aligned with NIST guidelines, optimizing tests and leveraging cumulative learning

## Data Availability

The dataset used for LLM training was accessed from https://www.cve.org/Downloads (accessed on 13 November 2024). The data generated and presented as part of this study can be consulted at https://github.com/AriadnaMoreno98/pentest-recommender-system-data (accessed on 13 November 2024).
